# On Self‐Love and Outgroup Hate: Opposite Effects of Narcissism on Prejudice via Social Dominance Orientation and Right‐Wing Authoritarianism[Fn per2114-note-1000]

**DOI:** 10.1002/per.2114

**Published:** 2017-08-04

**Authors:** Aleksandra Cichocka, Kristof Dhont, Arti P. Makwana

**Affiliations:** ^1^ School of Psychology University of Kent Canterbury UK

**Keywords:** narcissism, self‐esteem, authoritarianism, social dominance, prejudice

## Abstract

Previous research has obtained mixed findings as to whether feelings of self‐worth are positively or negatively related to right‐wing ideological beliefs and prejudice. We propose to clarify the link between self‐worth and ideology by distinguishing between narcissistic and non‐narcissistic self‐evaluations as well as between different dimensions of ideological attitudes. Four studies, conducted in three different socio‐political contexts: the UK (Study 1, N = 422), the US (Studies 2 and 3, Ns = 471 and 289, respectively), and Poland (Study 4, N = 775), investigated the associations between narcissistic and non‐narcissistic self‐evaluations, social dominance orientation (SDO), right‐wing authoritarianism (RWA), and ethnic prejudice. Confirming our hypotheses, the results consistently showed that after controlling for self‐esteem, narcissistic self‐evaluation was positively associated with SDO (accounting for RWA), yet negatively associated with RWA (accounting for SDO). These associations were similar after controlling for psychopathy and Machiavellianism (Study 3) as well as collective narcissism and Big Five personality characteristics (Study 4). Studies 2–4 additionally demonstrated that narcissistic self‐evaluation was indirectly positively associated with prejudice through higher SDO (free of RWA) but indirectly negatively associated with prejudice through lower RWA (free of SDO). Implications for understanding the role of self‐evaluation in right‐wing ideological attitudes and prejudice are discussed. Copyright © 2017 The Authors. European Journal of Personality published by John Wiley & Sons Ltd on behalf of European Association of Personality Psychology

Starting with the classic work of Adorno, Frenkel‐Brunswik, Levinson, and Sanford (1950), social scientists have sought to understand the links between socio‐political and intergroup attitudes and personality predispositions (see Cichocka & Dhont, in press; Duckitt & Sibley, 2010; Hodson & Dhont, 2015; Jost, Glaser, Kruglanski, & Sulloway, 2003; for reviews). A key line of inquiry in this research has linked political attitudes to self‐worth. As argued by Sniderman (1975) in his study of democratic politics, individual self‐evaluation ‘appears to lie at or near the center, rather than the periphery, of the personality system. It appears to be bound up with our most central needs and values, our conception of ourselves and others, our aspirations and our actions’ (p. 12). Indeed, several scholars have theorized that low feelings of self‐worth should be compensated by authoritarian attitudes, linked to right‐wing ideological inclinations and intolerance of others (Abrams & Hogg, 1988; Adorno et al., 1950; Sniderman & Citrin, 1971; Wilson, 1973).

Yet, after over 60 years of research, the evidence for this prediction remains mixed (Jost et al., 2003; Onraet, Van Hiel, & Dhont, 2013). Recent studies have even shown that exaggerated feelings of self‐worth (i.e. narcissism), rather than low feelings of self‐worth, are related to certain aspects of right‐wing ideological attitudes, especially to acceptance of inequality and preferences for group‐based hierarchy (e.g. Hodson, Hogg, & MacInnis, 2009; Zitek & Jordan, 2016). Remarkably, previous work has largely ignored the important distinction between narcissistic and non‐narcissistic 1In the literature, self‐evaluation without the narcissistic component is also sometimes referred to as secure, optimal, genuine, or mature self‐esteem (see e.g., Kernis, 2003). feelings of self‐worth (Kernis, 2003; Morf & Rhodewalt, 2001), which may show differential relations with right‐wing ideological beliefs. It is therefore important to disentangle the associations of these different forms of self‐evaluation with different dimensions of ideological attitudes. In the current research, we examine how narcissistic versus non‐narcissistic self‐evaluations are related to the dispositional key dimensions underpinning right‐wing belief systems: (i) acceptance of inequality—with social dominance orientation (SDO) as a typical indicator of this dimension, and (ii) maintenance of tradition and resistance to change—with right‐wing authoritarianism (RWA) as a typical indicator of this dimension (e.g. Altemeyer, 1998; Duckitt, 2001; Jost et al., 2003). Furthermore, we investigate the implications of these relations for ethnic prejudice.

## Self‐Evaluation and Political Ideology

Previous theorizing in the study of political ideology has suggested that stronger endorsement of right‐wing attitudes is linked to lower self‐evaluation (Jost et al., 2003; Sniderman & Citrin, 1971; Wilson, 1973). It was assumed that right‐wing attitudes associated with support for unequal social arrangements would compensate for low feelings of self‐worth by allowing people to attribute ‘weakness and incompetence to others’ (Sniderman & Citrin, 1971, p. 410). Nevertheless, empirical research has provided only weak support for this hypothesis. A meta‐analysis of 17 studies (total *N* = 1558) by Jost et al. (2003) showed that the relation between self‐esteem and ideology was significant and negative, yet relatively small (*r* = −.09, *p* < .001). 2Gignac and Szodorai (2016) recommended considering correlations of .10, .20, and .30 as small, typical, and relative large, respectively. However, we conducted power analyses using .14 as the indicator of a small effect to match the criterion for indirect effects analyses used by Fritz and MacKinnon (2007). These authors based their analyses on Cohen's (1988) criterion for a small effect explaining 2% of the variance. Across all text, we refer to correlations within the range of .10–.14 as weak/small. A more recent meta‐analysis by Onraet, Van Hiel, and Dhont (2013), based on a larger number of samples (51 studies, total *N* = 11,704), revealed that the overall relation was closer to zero and nonsignificant (*r* = −.02, *p* = .25). However, it is possible that the nature of this relation is more complex than it was originally presumed.

Remarkably, previous research and reviews focusing on the relation between self‐esteem and ideology rarely considered the distinction between different types of self‐evaluation. Yet, work on the psychology of self‐evaluation clearly indicates that non‐narcissistic feelings of self‐worth should be distinguished from narcissistic ones. Whereas self‐esteem reflects a generally positive evaluation of oneself, narcissism can be defined as an inflated self‐evaluation linked to feelings of entitlement and exploitativeness, grandiose exhibitionism, and the need to assert one's authority (Ackerman et al., 2011; see also Back et al., 2013; Kernis, 2003; Morf & Rhodewalt, 2001; Raskin & Terry, 1988). Because self‐esteem and narcissism both capture self‐confidence, they tend to be positively correlated. It is therefore important to account for their shared variance by analysing them simultaneously. This way, we can observe (i) *narcissistic self‐evaluation* (narcissism without the variance shared with self‐esteem), which captures the sense of uniqueness and exhibitionism, combined with feelings of entitlement and exploitativeness, and (ii) *non‐narcissistic self‐evaluation* (self‐esteem without the variance shared with narcissism), which captures unassuming pride in the self without the need for self‐enhancement or exploitation of others (Locke, 2009; Marchlewska & Cichocka, 2017; Paulhus, Robins, Trzesniewski, & Tracy, 2004). Narcissistic and non‐narcissistic self‐evaluations have unique social consequences. For example, while narcissism is positively associated with anti‐social behaviour and aggressiveness, non‐narcissistic self‐evaluation is *negatively* associated with these outcomes (Locke, 2009; Paulhus et al., 2004).

Ignoring the important distinction between narcissistic and non‐narcissistic self‐evaluation may explain why previous studies have not obtained solid or consistent evidence for the relation between self‐evaluations and ideology (Jost et al., 2003; Onraet, Van Hiel, & Dhont, 2013). Narcissism is considered a defensive type of self‐esteem (e.g. Baumeister, Bushman, & Campbell, 2000; Jordan, Spencer, Zanna, Hoshino‐Browne, & Correll, 2003), and narcissists are typically preoccupied with beliefs of entitlement and superiority over others (Campbell & Foster, 2007; Raskin & Terry, 1988). We propose that it is narcissistic self‐evaluation, rather than low self‐esteem, that should be linked to stronger endorsement of certain aspects of right‐wing attitudes. In fact, some early scholars suggested that conservatism is linked with self‐glorification and defensiveness, accompanied by being critical of others (Brown, 1965; McClosky, 1958), all of which are characteristic for narcissism. Furthermore, in line with this theorizing, Adorno et al. (1950/2010) have suggested that true liberals rarely are narcissistic.

Empirical evidence also indicates that right‐wing ideological beliefs are associated with narcissistic, defensive self‐evaluation. For instance, in a sample of elderly Belgians, Van Hiel and Brebels (2011) found that conservatism was positively correlated with narcissism, and this correlation was stronger than the positive correlation of conservatism with self‐esteem (for narcissism *r* = .36, for self‐esteem *r* = .19, correlation difference test 3We computed the correlation differences based on Steiger's (1980) formula (Lee & Preacher, 2013).
*z* = −2.44, *p* = .014). Furthermore, in a study conducted by Soenens and Duriez (2012), conservatism was positively associated with contingent self‐esteem—a type of self‐evaluation that is, similarly to narcissism, associated with defensiveness (Kernis, Lakey, & Heppner, 2008; Kernis & Paradise, 2002), but was not significantly associated with non‐contingent self‐esteem (for contingent self‐esteem *r* = .29, *p* < .01, for non‐contingent self‐esteem *r* = .11, ns; correlation difference test *z* = −1.98, *p* < .05). These findings suggest that high narcissistic, rather than low non‐narcissistic self‐evaluation, might be more strongly associated with right‐wing attitudes.

## Narcissism and Different Dimensions of Right‐Wing Ideologies

Narcissists may find certain elements of right‐wing ideological systems more appealing than others. Researchers often distinguish between two related but distinct dimensions of right‐wing belief systems expressing different motivational goals (Altemeyer, 1998; Duckitt, 2001; Jost et al., 2003; Sibley & Duckitt, 2008; Weber & Federico, 2007). The first dimension pertains to the idea that inequality is inevitable (or even desirable) and expresses underlying motivational goals of superiority and power over others. It is typically captured by SDO, defined as a general desire for group‐based social hierarchy and inequality between social groups (Pratto, Sidanius, Stallworth, & Malle, 1994; Sidanius & Pratto, 1999). The second dimension pertains to resistance to change and expresses the motivational goal of maintaining traditional social arrangements, order, and stability. This second dimension is captured by RWA, defined as the endorsement of traditional social norms and values (i.e. conventionalism), authoritarian submission, and authoritarian aggression (Altemeyer, 1981). SDO and RWA have been shown to be complementary predictors of a wide range of outcome variables in the political and intergroup domains, including, but not limited to, ethnic prejudice, sexist beliefs, and homophobia (Ekehammar, Akrami, Gylje, & Zakrisson, 2004; Sibley & Duckitt, 2008; Meeusen & Dhont, 2015; Van Hiel & Mervielde, 2005).

Because those high in narcissistic self‐evaluation are motivated to validate their self‐image by establishing their power and dominance over others (Bushman & Baumeister, 1998; Raskin, Novacek, & Hogan, 1991; see also Leckelt, Küfner, Nestler, & Back, 2015), they should be particularly attracted to the ideological values and beliefs expressed by SDO. For example, narcissistic self‐evaluation tends to be contingent on meeting standards associated with competition, but not those associated with other domains, such as family, religion, or being a virtuous person (Zeigler‐Hill, Clark, & Pickard, 2008). Furthermore, narcissists tend to perceive themselves as superior to others on traits reflecting their high competence (e.g. in terms of their intellectual skills) rather than on those reflecting their morality (Campbell, Rudich, & Sedikides, 2002). Indeed, several authors have reported a positive correlation between narcissism and SDO (Carnahan & McFarland, 2007; Golec de Zavala, Cichocka, & Iskra‐Golec, 2013; Hodson et al., 2009; Zitek & Jordan, 2016). Therefore, we expected that narcissism would be positively related to greater endorsement of ideological attitudes associated with power and dominance (indicated by SDO), and that this effect should be most pronounced when we observe narcissistic self‐evaluation (i.e. controlling for self‐esteem). We expected a different pattern for the association between narcissism and RWA. Right‐wing authoritarians value social arrangements that guard moral standards and social cohesion as opposed to individual freedom and self‐expression (Duckitt & Sibley, 2010). These values are in contrast with the narcissistic sense of uniqueness. Narcissists like to see themselves as rebellious and non‐conforming (Raskin & Terry, 1988) as well as open to new experiences and creative (Goncalo, Flynn, & Kim, 2010). They also tend to be less pre‐occupied with morality than status (Campbell et al., 2002). In past studies, the associations between narcissism and RWA have been inconsistent (Golec de Zavala, Cichocka, & Iskra‐Golec, 2013; Hodson et al., 2009). However, these studies did not consider narcissism and self‐esteem as joined predictors of RWA. We expected that narcissism would be negatively associated with RWA, especially when examining the associations with narcissistic self‐evaluation (i.e. controlling for self‐esteem).

We further expected that any associations between narcissistic self‐evaluation and ideological attitudes would be more pronounced if we considered the overlap between SDO and RWA. SDO and RWA seem to overlap especially in their acceptance for intergroup aggressiveness and, thus, are typically positively correlated (e.g. Asbrock, Sibley, & Duckitt, 2010; Dhont & Hodson, 2014; Ekehammar et al., 2004; Kandler, Bell, & Riemann, 2016). In order to observe unique relations for RWA and SDO, it is important to account for their shared variance. Once the variance shared with SDO is co‐varied out from RWA, we should better capture the maintenance of tradition and resistance to change component of ideology. Once the variance shared with RWA is co‐varied out from SDO, we should better capture the acceptance of inequality component of ideology (e.g. Jost et al., 2003; for a similar argument see Dhont, Hodson, & Leite, 2016). Therefore, we tested the opposing relation between narcissistic self‐evaluation and SDO versus RWA after accounting for the variance shared between these two dimensions of right‐wing ideological beliefs.

## Overview of the Current Research

The current set of studies tested two key hypotheses. First, we hypothesized that narcissistic self‐evaluation (i.e. narcissism without the variance shared with self‐esteem) would be positively associated with SDO free of RWA (Hypothesis 1). Second, we hypothesized that narcissistic self‐evaluation would be negatively associated with RWA free of SDO (Hypothesis 2). We tested these hypotheses in four studies conducted in three different socio‐political contexts (the UK, the USA, and Poland). In each study, we examined the associations of narcissism with the two dimensions of ideological attitudes: SDO and RWA. To disentangle the complex relations between the variables, we accounted for the variance shared between narcissism and self‐esteem, as well between RWA and SDO in all studies. 4In the Online Supplement 1 available on the Open Science Framework (https://osf.io/xscvw/), we have included additional results to test the associations of narcissism (without controlling for self‐esteem, Table S1) and self‐esteem (without controlling for narcissism, Table S2) with SDO (free of RWA) and RWA (free of SDO), as well as the associations between narcissistic and secure self‐evaluation with RWA and SDO (without controlling for the shared variance between RWA and SDO, Table S3).


In Studies 2–4, we aimed to replicate and extend the findings by testing the indirect relations of narcissistic self‐evaluation with ethnic prejudice via the mediating role of SDO (free of RWA) and RWA (free of SDO). To date, only a few studies have investigated the relations between narcissism and ethnic prejudice, yielding mixed results. Some studies reported significant positive relations between narcissism and prejudice or ethnocentrism (e.g. Bizumic & Duckitt, 2008; Hodson et al., 2009), whereas others obtained rather weak or non‐significant relations (e.g. McFarland, 2010; Schnieders & Gore, 2011). Following our hypotheses that narcissistic self‐evaluation is expected to be differentially related to SDO (free of RWA) and RWA (free of SDO), we did not expect to find a straightforward, positive relation between narcissistic self‐evaluation and prejudice. Instead, we specifically tested whether narcissistic self‐evaluation is indirectly related to ethnic prejudice through differential and opposing mediating pathways of SDO and RWA. Given the mixed findings obtained in past research (Jost et al., 2003; Onraet, Van Hiel, & Dhont, 2013), we did not have specific predictions about the association between non‐narcissistic self‐evaluation (i.e. self‐esteem controlling for narcissism) and ideological attitudes.

In Studies 3 and 4, we tested the robustness of these associations by additionally controlling for related individual difference variables that could possibly confound our findings. Specifically, in Study 3, we controlled for psychopathy and Machiavellianism to account for their overlap with narcissism (Hodson et al., 2009; Paulhus & Williams, 2002). In Study 4, we controlled for collective narcissism (Golec de Zavala, Cichocka, Eidelson, & Jayawickreme, 2009) as well for the basic Big Five personality traits (McCrae & Costa, 1987). Finally, in all studies we checked the analyses controlling for gender, which is robustly associated with both narcissism (with higher levels of narcissism observed among men than women; Grijalva et al., 2015) and ideological attitudes, especially SDO (with higher levels of SDO observed among men than women; e.g. Lippa & Arad, 1999; Sidanius & Pratto, 1999). 5A summary of separate analyses for men and women is included in the supplementary results (Online Supplement 1, Tables S5.1–S5.7 available at https://osf.io/xscvw/). The measures and datasets of all four studies are available online on the project page on the website of the Open Science Framework (https://osf.io/xscvw/).

## Study 1

Study 1 was conducted in the UK and was designed to test the hypotheses that narcissism shows differential relations with two right‐wing ideological dimensions. We expected that narcissistic self‐evaluation would be positively related to SDO (free of RWA; Hypothesis 1) and negatively related to RWA (free of SDO; Hypothesis 2).

## Method

### Participants

Undergraduate students at a UK university took part in an online mass test which included measures of narcissism, self‐esteem, RWA, and SDO. These measures were completed by 422 participants, including 350 females, 49 males, and 23 students who did not indicate their gender. With this samples size, we achieved a power of .83 to detect a small effect (*β* = .14; two‐tailed) for narcissism as predictor. The mean age of the sample was *M* = 19.67 years (*SD* = 3.88).

### Measures

Narcissism was measured with the Narcissistic Personality Inventory (NPI; Raskin & Hall, 1979). Participants were presented with 40 pairs of diagnostic and non‐diagnostic statements and were asked to choose the ones that describe them best (e.g. ‘The thought of ruling the world frightens the hell out of me’ versus ‘If I ruled the world it would be a better place’), α = .85, *M =* .29, *SD =* .16.

Self‐esteem was measured with Rosenberg's (1965) self‐esteem scale. Participants responded to the 10 items (e.g. ‘On the whole I am satisfied with myself’) on a scale from 1 = *strongly disagree* to 5 = *strongly agree*, α = .91, *M =* 4.52, *SD =* 1.21.

Right‐wing authoritarianism was measured with 12 items of Duckitt, Bizumic, Krauss, and Heled's (2010) scale (e.g. ‘What our country needs most is discipline, with everyone following our leaders in unity’). Participants indicated to what extent they agree with the statements on a scale from 1 = *strongly disagree* to 7 = *strongly agree*, α = .78, *M* = 3.47, *SD* = 0.84.

Social dominance orientation was measured with the 16‐item SDO scale (e.g. ‘Inferior groups should stay in their place’; Pratto et al., 1994). Participants indicated to what extent they agree with the statements on a scale from 1 = *strongly disagree* to 7 = *strongly agree*, α = .92, *M* = 2.33, *SD* = 0.98.

## Results

First, we calculated the zero‐order correlations between the variables (Table 1). Narcissism was significantly positively related to SDO but was not significantly related to RWA. Self‐esteem was weakly, yet significantly and positively related to RWA.

**Table 1:** Zero‐order correlations between manifest variables (Study 1)

	1	2	3
1. Narcissism	—		
2. Self‐esteem	.39***	—	
3. RWA	−.07	.12*	—
4. SDO	.16***	.07	.38***

* *p* < .05.

*** *p* < .001.

Next, we tested the hypotheses by conducting structural equation modelling (SEM) with latent variables using robust maximum likelihood estimation in Mplus (version 7.2, Muthén & Muthén, 1998–2014). To smooth measurement error and maintain an adequate ratio of cases‐to‐parameters, we averaged item subsets into five balanced indicator parcels for the latent factor of narcissism and three balanced indicator parcels for the latent factors of self‐esteem, SDO, and RWA. 6Parcels were computed based on item‐to‐construct loadings (see Online Supplement 2 for details https://osf.io/xscvw/). The measurement model showed a good model fit, *χ* ^2^(71) = 147.84, *p* < .001; *RMSEA* = .051; *SRMR* = .037; *CFI* = .975.

To investigate the unique associations of narcissistic and non‐narcissistic self‐evaluation with SDO and RWA while accounting for the overlap between SDO and RWA, we tested two latent models. Both models included paths from narcissistic and non‐narcissistic self‐evaluation to SDO and RWA. In the first model, we additionally included a path from RWA to SDO in order to test the hypothesis that narcissistic self‐evaluation would be positively related to the dominance aspect of right‐wing attitudes, and thus the SDO‐scores (free of RWA). The second model included a path from SDO to RWA to test the hypothesized negative relation between narcissistic self‐evaluation and the social conventionalism aspect of right‐wing attitudes, and thus the RWA scores (free of SDO).

As expected, the results of these models showed that narcissistic self‐evaluation and SDO (free of RWA) were positively associated, whereas narcissistic self‐evaluation and RWA (free of SDO) were negatively associated (see Table 2). Furthermore, the opposite pattern of results was observed for non‐narcissistic self‐evaluations, showing negative associations with SDO (free of RWA) and positive associations with RWA (free of SDO). The effects were very similar when we controlled for gender.

**Table 2:** Results (standardized estimates) of latent models testing the associations of narcissistic and non‐narcissistic self‐evaluations with SDO (free of RWA) and RWA (free of SDO; Studies 1–4) and ethnic prejudice (Studies 2–4)

	Study 1		Study 2		Study 3		Study 4	
Associations	*β* [95% CIs]	*p*	*β* [95% CIs]	*p*	*β* [95% CIs]	*p*	*β* [95% CIs]	*p*
Narc – Self‐esteem	.426 [.334, .518]	<.001	.296 [.205, .387]	<.001	.190 [.063, .316]	.003	.104 [.034, .174]	.004
Narc SE ➔ SDO_f_	.275 [.159, .392]	<.001	.402 [.297, .506]	<.001	.455 [.354, .556]	<.001	.086 [.020, .152]	.011
Non‐Narc SE ➔ SDO_f_	−.111 [−.219, −.004]	.042	−.145 [−.243, −.046]	.004	−.253 [−.356, −.150]	<.001	.067 [.001, .132]	.046
Narc SE ➔ RWA_f_	−.273 [−.389, −.156]	<.001	−.144 [−.256, −.031]	.013	−.275 [−.416, −.135]	<.001	−.150 [−.214, −.085]	<.001
Non‐Narc SE ➔ RWA_f_	.203 [.075, .330]	.002	.222 [.115, .329]	<.001	.312 [.187, .436]	<.001	−.036 [−.101, .029]	.279
Narc SE ➔ Prejudice	/	/	−.004 [−.096, .089]	.940	−.076 [−.206, .054]	.254	−.018 [−.085, .048]	.590
Non‐Narc SE ➔ Prejudice	/	/	.067 [−.016, .150]	.115	−.057 [−.162, .047]	.282	−.141 [−.205, −.076]	<.001
SDO_f_ ➔ Prejudice	/	/	.508 [.412, .603]	<.001	.699 [.574, .823]	<.001	.280 [.213, .348]	<.001
RWA_f_ ➔ Prejudice	/	/	.327 [.230, .425]	<.001	.158 [.034, .281]	.013	.180 [.110, .250]	<.001

*Note:* Narc SE = Narcissistic self‐evaluation; Non‐narc SE = Non‐narcissistic self‐evaluation. The paths to SDO_f_ and RWA_f_ were calculated in two separate models which allowed us to estimate the associations for SDO, accounting for RWA (i.e. by including the path from RWA to SDO) and for RWA, accounting for SDO (by including the path from SDO to RWA).

In sum, the findings of Study 1 confirmed Hypothesis 1 stating that narcissistic self‐evaluation was positively associated with SDO. As expected, this relation becomes more pronounced after co‐varying out the variance shared with RWA, confirming that it is particularly the dimension of right‐wing ideologies related to acceptance of inequality (relevant to dominance strivings and hierarchy) that is associated with narcissistic self‐evaluation. Further, in line with Hypothesis 2, we found a negative relation between narcissism and RWA, once we co‐varied out the variance shared with SDO. This finding indicates that narcissistic people tend to dislike the aspect of right‐wing ideologies reflecting resistance to change and preservation of traditional social norms, and rather value individual freedom and self‐expression. Noteworthy, whereas narcissistic self‐evaluation showed a negative relation with RWA (free of SDO), non‐narcissistic self‐evaluation was significantly, positively related to RWA (free of SDO).

## Study 2

Study 1 provided supporting evidence for both hypotheses regarding the differential relations of narcissistic self‐evaluation with SDO and RWA, after their shared variance was controlled for. The aims of the next studies were twofold. First, we aimed to replicate the findings of Study 1 (a large student sample from the UK) in a sample of adults from a different context (the USA). Second, we aimed to extend the findings of Study 1 by testing whether these two ideological dimensions might underlie the relation between narcissism and prejudice. Raskin and Hall (1981) originally described narcissistic individuals as not only self‐aggrandising and self‐absorbed, but also lacking in empathy. Indeed, research has demonstrated that narcissism is associated with anti‐social tendencies, including interpersonal aggressiveness (e.g. Back et al., 2013; Bushman & Baumeister, 1998; Donnellan, Trzesniewski, Robins, Moffitt, & Caspi, 2005; Locke, 2009) and negative perceptions of humanity (Cichocka, Marchlewska, & Golec de Zavala, 2016). McFarland (2010) argued that the lack of concern for others demonstrated by narcissists may lead them to display higher levels of generalized prejudice. Yet, the evidence for this relation is mixed.

Narcissism was found to be positively related to anti‐immigrant prejudice in a Canadian student sample (Hodson et al., 2009) as well as to prejudice towards some (but not all) ethnic minority groups in a student sample from New Zealand (Bizumic & Duckitt, 2008). However, McFarland (2010) did not find narcissism to be a significant predictor of prejudice (over and above other predictors including RWA and SDO) in two samples of American students and adults. Schnieders and Gore (2011) reported a non‐significant correlation between narcissism and anti‐immigrant prejudice in the US. None of these previous studies have considered the possibility that narcissism may show differential indirect relations with prejudice through different mediating mechanisms.

More specifically, in line with idea that narcissists want to display their superiority beliefs and dominance strivings, we expected that narcissism would be positively, indirectly related to outgroup prejudice via the mediating role of SDO, once we control for self‐esteem and RWA (Hypothesis 3). However, narcissism might also be negatively, indirectly related to prejudice via RWA, once we control for self‐esteem and SDO (Hypothesis 4), to the extent that prejudicial beliefs might be considered a way of preserving social traditions and ingroup cohesion. These opposing indirect associations should result in an overall weak or inconsistent link between narcissism and ethnic prejudice. Even though we test these indirect effects in mediation models, they should be interpreted with caution. Given the cross‐sectional nature of our samples, these models do not test for the causal relations between the variables; rather, they indicate where the complex nature of the associations might stem from.

## Method

### Participants

We conducted a survey among 516 Mturk workers located in the US. Participants filled out measures of self‐esteem, narcissism (counterbalanced), followed by measures of right‐wing authoritarianism, social dominance orientation (counterbalanced), and ethnic prejudice. 7Study 2 also included additional measures reported in Cichocka, Marchlewska et al. (2016), who focused on the links between narcissism and self‐esteem with conspiracy beliefs, but did not examine RWA, SDO or prejudice. Because we examined prejudice against Black people, analyses excluded 44 participants who reported their racial background as Black or multi‐racial (Black and White) as well as one person who failed to report their racial background. The final sample included 471 participants including 278 females and 193 males, aged 18–75 (*M* = 35.51, *SD* = 13.31). With this samples size, we achieved a power of .87 to detect a small effect for narcissism as predictor. The sample was also large enough to detect even small indirect effect paths (i.e. multiplication of small ‘A‐paths’—from narcissism to SDO/RWA and small ‘B‐paths’ from SDO/RWA to prejudice) with bias‐corrected bootstrapping with the power of .80 (Fritz & MacKinnon, 2007).

### Measures

Narcissism (α = .88, *M =* .30, *SD =* .19), RWA (α = .88, *M* = 3.66, *SD* = 1.20), and SDO (α = .95, *M* = 2.45, *SD* = 1.26) were measured with the same scales as used in Study 1. We also used the same self‐esteem items as in Study 1, but these were completed on a 5‐point (rather than 7‐point) scale (1 = *strongly disagree*; 5 = *strongly agree,* α = .93, *M =* 3.76, *SD =* 0.92). Prejudice was measured with 4 items measuring subtle prejudice towards Black people (e.g. ‘Blacks living here should not push themselves where they are not wanted’; Pettigrew & Meertens, 1995). Participants indicated to what extent they agree with the statements on a scale from 1 = *strongly disagree* to 7 = *strongly agree*, α = .85, *M* = 3.16, *SD* = 1.57.

## Results and Discussion

The zero‐order correlations showed that narcissism was significantly positively correlated with SDO and prejudice*,* but was not significantly correlated with RWA. Self‐esteem showed a significant positive correlation with both RWA and prejudice but was not significantly correlated with SDO. Both RWA and SDO were significantly positively correlated with prejudice (Table 3).

**Table 3:** Zero‐order correlations between manifest variables (Study 2)

	1	2	3	4
1. Narcissism	—			
2. Self‐esteem	.26***	—		
3. RWA	.05	.18***	—	
4. SDO	.35***	.03	.29***	—
5. Prejudice	.22***	.11*	.44***	.60***

* *p* < .05.

*** *p* < .001.

**Figure 1:**
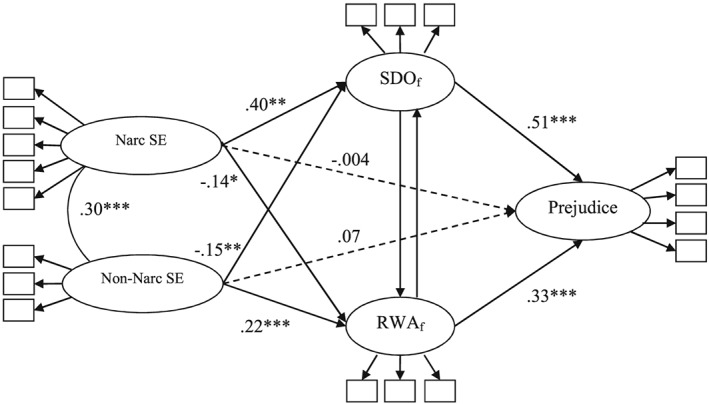
Results of latent models in Study 2 testing the effects (standardized estimates) of narcissistic and non‐narcissistic self‐evaluation on prejudice via SDO (free of RWA) and RWA (free of SDO). *Note.* Narc SE = Narcissistic self‐evaluation; Non‐narc SE = Non‐narcissistic self‐evaluation. The results were obtained from two separate models in order to estimate the associations for SDO_f_ = SDO accounting for RWA (i.e. including the path from RWA to SDO) and for RWA_f_ = RWA accounting for SDO (i.e. including the path from SDO to RWA). * *p* < .05. ** *p* < .01. *** *p* < .001. Dashed arrows represent non‐significant paths.

Next, we tested our hypotheses using SEM with latent variables, following similar analytic procedures as in Study 1. We averaged item subsets into five balanced indicator parcels for the latent factor of narcissism and three balanced indicator parcels for the latent factors of self‐esteem, SDO, and RWA. 8See Online Supplement 2 (https://osf.io/xscvw/) for the details of the parcels. The four prejudice items served as indicators for the latent prejudice factor. The measurement model showed a good model fit, *χ* ^2^(125) = 331.18, *p* < .001; *RMSEA* = .059; *SRMR* = .050; *CFI* = .963. As in Study 1, we tested two models to investigate the unique associations of narcissistic and non‐narcissistic self‐evaluations with SDO after controlling for RWA, and with RWA after controlling for SDO, respectively. We further extended the two models by adding prejudice as the criterion variable and including paths from the latent variables representing narcissism, self‐esteem, SDO, and RWA to prejudice. This allowed us to test our differential mediation hypotheses stating that narcissistic self‐evaluation is *positively* indirectly related to ethnic prejudice through SDO (free of RWA), yet *negatively* indirectly related to prejudice through RWA (free of SDO). The results of these analyses are presented in Figure 1 and Table 2.

In line with Hypotheses 1 and 2 and replicating the findings of Study 1, we observed a significant positive relation between narcissistic self‐evaluation and SDO (free of RWA) and a significant, negative relation between narcissistic self‐evaluation and RWA (free of SDO) (Table 2). Also consistent with the findings from Study 1, non‐narcissistic self‐evaluation was significantly, positively related to RWA (free of SDO) and significantly negatively related to SDO (free of RWA). Furthermore, RWA (free of SDO) and SDO (free of RWA) were significantly positively related to prejudice, whereas the direct paths from narcissistic and non‐narcissistic self‐evaluations were non‐significant.

Next, we examined the indirect associations of narcissistic and non‐narcissistic self‐evaluations with prejudice via SDO (free of RWA) and RWA (free of SDO). In line with Hypotheses 3 and 4, the results of these analyses confirmed the positive indirect association between narcissistic self‐evaluation and prejudice via SDO (free of RWA), indirect effect = 1.346 [0.907, 1.930] 9Square brackets for the indirect effects indicate 95% bias‐corrected bootstrap confidence intervals based on 10,000 bootstrap samples. (standardized estimate = .204), and a significant negative indirect association between narcissistic self‐evaluation and prejudice through RWA (free of SDO), indirect effect = −0.310 [−0.594, −0.069] (standardized estimate = −.047). The indirect associations between non‐narcissistic self‐evaluation and prejudice through SDO (free of RWA) and RWA (free of SDO) showed the opposite pattern, with a negative indirect effect through SDO (free of RWA), indirect effect = −0.099 [−0.172, −0.036] (standardized estimate = −.073), and a positive indirect effect through RWA (free of SDO), indirect effect = 0.097 [0.046, 0.165] (standardized estimate = .073). All direct and indirect effects remained significant after controlling for gender. Overall, these results confirmed the differential mediation hypothesis stating that narcissistic self‐evaluation is indirectly positively associated with ethnic prejudice via SDO (free of RWA) but indirectly negatively associated with prejudice via RWA (free of SDO).

## Study 3

The aim of Study 3 was twofold. First, we sought to examine whether the effects for prejudice will extend beyond the context of anti‐Black sentiments. Therefore, in Study 3, which was conducted in the US, we included a wider range of prejudice indicators tapping into attitudes towards several ethnic outgroups. Second, we tested whether the effects of narcissistic and non‐narcissistic self‐evaluations on ideology and prejudice would still hold after controlling for psychopathy and Machiavellianism, two concepts that are strongly associated with narcissism (Paulhus & Williams, 2002) and contribute to anti‐social attitudes and behaviours, including prejudice (for a review see Furnham, Richards, & Paulhus, 2013). Specifically, Hodson et al. (2009) found that besides narcissism, psychopathy, and Machiavellianism were also correlated with higher SDO and prejudice. Therefore, in Study 3, we added measures of Machiavellianism and psychopathy to examine whether narcissistic self‐evaluation would be a unique predictor of ideology and prejudice over and above these possible confounding variables.

## Method

### Participants

We conducted a survey among 301 Mturk workers located in the US who completed measures of self‐esteem, narcissism, psychopathy, and Machiavellianism, followed by measures of RWA, SDO, and ethnic prejudice. Twelve participants were excluded from the analyses because they belonged to an ethnic minority group. The final sample included 289 participants, 171 females and 118 males, aged 19–75 (*M* = 37.93, *SD* = 12.68). This sample size gave us a power of .68 to detect small effects, so the study can be considered slightly underpowered. The study was also underpowered to detect indirect effects with a power of .80 (Fritz & MacKinnon, 2007). 10The required sample size would be 380, even when assuming a small effect for the A‐paths (from self‐evaluations to ideological variables) and a medium (rather than small) effect for the B‐paths (from the ideological variables to prejudice).


### Measures

Narcissism was measured with a simplified version (Ang & Yusof, 2006; see Grosz et al., in press) of the 40‐item NPI (Raskin & Hall, 1979). Participants rated to what extent statements representing narcissistic traits described them on a scale from 1 = *strongly disagree* to 7 = *strongly agree*, α = .96, *M =* 3.68, *SD =* 1.01.

Machiavellianism and psychopathy were measured with nine items each from the Short Dark Triad subscales (Jones & Paulhus, 2014). Participants were asked to respond to the statements on a scale from 1 = *strongly disagree* to 7 = *strongly agree*. A sample statement for Machiavellianism reads ‘Most people can be manipulated’, α = .87, *M =* 4.03, *SD =* 1.18. A sample statement for psychopathy reads ‘People who mess with me always regret it’, α = .83, *M =* 2.35, *SD =* 1.02.

Participants also completed the same measures of self‐esteem (α = .93, *M =* 5.32, *SD =* 1.28), RWA ***(***α = .92, *M* = 3.80, *SD* = 1.37), and SDO (α = .96, *M* = 2.43, *SD* = 1.34) as in Studies 1 and 2.

To measure ethnic prejudice, we used four indicators. Where needed, the items were recoded so that higher scores reflect greater ethnic prejudice. First, respondents indicated their general attitude towards the following ethnic (or religious) outgroups: Black people, South Asian people, ethnic minorities, immigrants, Muslims, and Latinos on attitude thermometers ranging from 0 to 10° = *extremely unfavourable* to 91–100° = *extremely favourable*, α = .92; *M* = 3.82; *SD* = 1.92 (see also Dhont, Hodson, Costello, & MacInnis, 2014). The second prejudice indicator consisted of three social distance items (see Bogardus, 1933) asking participants how much would it bother them ‘to marry someone from an ethnic minority background, or have someone in your family do so’; ‘to have someone from an ethnic minority background as a doctor’, and ‘to have people from ethnic minority backgrounds as neighbors on the same street’ (1 = *not at all* to 7 = *very much;* α = .86; *M* = 1.69; *SD* = 1.20). The third indicator consisted of four bipolar scales (α = .97; *M* = 2.47; *SD* = 1.37) asking respondents to describe how they generally feel towards African Americans (1–7; cold–warm, negative–positive, hostile–friendly, contempt–respect; Wright, Aron, McLaughlin‐Volpe, & Ropp, 1997). Finally, the fourth indicator consisted of four items of the subtle prejudice scale (α = .84; *M* = 3.11; *SD* = 1.55, e.g. ‘African Americans living here should not push themselves where they are not wanted’; based on Pettigrew & Meertens, 1995). These items were rated on 7‐point scales (1 = *strongly disagree* to 7 = *strongly agree*). Because the four indicators were highly correlated, we created one single index of ethnic prejudice (α = .93, i.e. one average score as a manifest variable, and one latent factor in the SEM analyses).

## Results and Discussion

The zero‐order correlations showed that narcissism was significantly positively correlated with SDO but was not significantly related to RWA and prejudice (Table 4). Self‐esteem was significantly positively correlated with RWA but not with SDO and prejudice. Both RWA and SDO were significantly positively correlated with prejudice. As expected, psychopathy and Machiavellianism were also significantly related to most of the key variables, confirming the importance of including them as statistical controls. Both variables showed positive correlations with narcissism, SDO, and prejudice, and negative correlations with self‐esteem. Psychopathy, but not Machiavellianism, was also significantly negatively related to RWA.

**Table 4:** Zero‐order correlations between manifest variables (Study 3)

	1	2	3	4	5	6
1. Narcissism	—					
2. Psychopathy	.45***	—				
3. Machiavellianism	.44***	.54**	—			
4. Self‐esteem	.18**	−.36**	−.22**	—		
5. SDO	.37***	.35**	.34**	−.06	—	
6. RWA	−.01	−.16**	−.07	.21**	.36**	—
7. Prejudice	.15***	.34***	.32***	−.09	.63***	.32***

** *p* < .01.

*** *p* < .001.

Similar to Study 2, we then tested latent models to investigate the paths from the latent factors of narcissism and self‐esteem (indicated by five and three parcels, respectively) to the latent factors of SDO and RWA, each indicated by three parcels. 11See Online Supplement 2 (https://osf.io/xscvw/) for details of the parcels. We also included paths from these key variables to a latent prejudice factor to test our differential mediation hypothesis. The scores of the four prejudice indicators served as indicators for the prejudice factor. The fit of the measurement model was acceptable, *χ* ^2^(125) = 321.26, *p* < .001; *RMSEA* = .074; *SRMR* = .057; *CFI* = .953.

**Figure 2:**
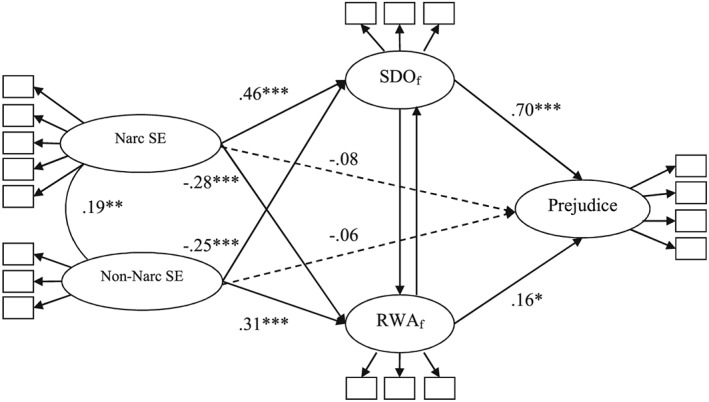
Results of latent models in Study 3 testing the effects (standardized estimates) of narcissistic and non‐narcissistic self‐evaluation on prejudice via SDO (free of RWA) and RWA (free of SDO). *Note.* Narc SE = Narcissistic self‐evaluation; Non‐narc SE = Non‐narcissistic self‐evaluation. The results were obtained from two separate models in order to estimate the associations for SDO_f_ = SDO accounting for RWA (i.e. including the path from RWA to SDO) and for RWA_f_ = RWA accounting for SDO (i.e. including the path from SDO to RWA). * *p* < .05. ** *p* < .01. *** *p* < .001. Dashed arrows represent non‐significant paths.

As expected, the results showed a significant positive relation between narcissistic self‐evaluations and SDO (free of RWA), confirming Hypothesis 1, and a significant negative relation between narcissistic self‐evaluation and RWA (free of SDO), confirming Hypothesis 2 (see Table 2 and Figure 2). The reverse pattern of results was found for non‐narcissistic self‐evaluation, showing a significant negative relation with SDO (free of RWA), yet a significant positive relation with RWA (free of SDO). Replicating the results of Study 2, RWA (free of SDO) and SDO (free of RWA) were, in turn, significantly positively related to prejudice, whereas the direct paths from narcissistic and non‐narcissistic self‐evaluations were non‐significant.

Testing the indirect relations of narcissistic and non‐narcissistic self‐evaluations with prejudice via SDO (free of RWA) and RWA (free of SDO) confirmed Hypotheses 3 and 4. We found a positive indirect association between narcissistic self‐evaluation and prejudice via SDO (free of RWA), indirect effect = −.315 [.228, .430] (standardized estimate = .318), and a negative, yet weak, indirect association between narcissistic self‐evaluation and prejudice through RWA (free of SDO), indirect effect = −.043 [−.098, −.010] (standardized estimate = −.043). Furthermore, we found a negative indirect association between non‐narcissistic self‐evaluation and prejudice through SDO (free of RWA) indirect effect = −.132 [−.200, −.081] (standardized estimate = −.177), and a positive, indirect association between non‐narcissistic self‐evaluation and prejudice through RWA (free of SDO), indirect effect = .037 [.009, .081] (standardized estimate = .049).

Next, we tested these same associations after including psychopathy, Machiavellianism and gender as statistical controls, *χ* ^2^(252) = 596.230, *p* < .001; *RMSEA* = .069; *SRMR* = .068; *CFI* = .938. Overall, the pattern of results remained fairly similar, although the strength of the associations was somewhat weaker after adding psychopathy, Machiavellianism, and gender to the model (see Table 5). The positive association between narcissistic self‐evaluation and SDO (free of RWA) was still significant and sizeable. Narcissistic self‐evaluation also still showed a negative association with RWA (free of SDO). Although this effect was in the small to moderate range, it became non‐significant, which might be due to the low statistical power of the study. Furthermore, the negative association between non‐narcissistic self‐evaluation and SDO (free of RWA) was not significant, whereas the positive association of non‐narcissistic self‐evaluation with RWA (free of SDO) remained significant.

**Table 5:** Results (standardized estimates) of latent models testing the associations of narcissistic and non‐narcissistic self‐evaluations with SDO (free of RWA), RWA (free of SDO) and ethnic prejudice, controlling for psychopathy, Machiavellianism, and gender (Study 3)

	SDO_f_		RWA_f_		Ethnic prejudice	
	*β* [95% CIs]	*p*	*β* [95% CIs]	*p*	*β* [95% CIs]	*p*
Narc SE	.240 [.082, .398]	.003	−.172 [−.347, .003]	.054	−.278 [−.456, −.099]	.002
Non‐Narc SE	−.106 [−.244, .032]	.134	.236 [.073, .399]	.005	.094 [−.038, .204]	.161
Psychopathy	.203 [.027, .380]	.024	−.135 [−.330, .061]	.177	.225 [.020, .431]	.032
Machiavellianism	.140 [−.022, .301]	.090	−.012 [−.181, .157]	.891	.198 [.050, .345]	.009
Gender (1 = M, 0 = F)	.122 [.021, .223]	.018	−.247 [−.349, −.146]	<.001	.070 [−.042, .177]	.227
SDO_f_					.583 [.437, .705]	<.001
RWA_f_					.233 [.105, .340]	<.001

*Note:* Narc SE = Narcissistic self‐evaluation; Non‐narc SE = Non‐Narcissistic Self‐Evaluation. The paths to SDO_f_ and RWA_f_ were calculated in two separate models which allowed us to estimate the associations for SDO, accounting for RWA (i.e. by including the path from RWA to SDO) and for RWA, accounting for SDO (i.e. by including the path from SDO to RWA).

With respect to the indirect associations, after adjusting for psychopathy, Machiavellianism and gender, we found a positive indirect effect of narcissistic self‐evaluations on prejudice via SDO (free of RWA), indirect effect = .139 [.049, .255] (standardized estimate = .140). Also, the negative indirect effect of narcissistic self‐evaluation on prejudice via RWA (free of SDO) remained significant, indirect effect = −.040 [−.101, −.002] (standardized estimate = −.041). Furthermore, the indirect effect of non‐narcissistic self‐evaluation on prejudice via SDO (free of RWA) was no longer significant, indirect effect = −.046 [−.117, .011] (standardized estimate = −.062), whereas the indirect effect of non‐narcissistic self‐evaluation on prejudice via RWA (free of SDO), was still positive and significant, indirect effect = .041 [.012, .089] (standardized estimate = .056). Overall, even after controlling for psychopathy and Machiavellianism, the pattern of results was consistent with the findings of Studies 1 and 2.

## Study 4

In Study 4, we investigated whether our effects would extend beyond the context of Western, Capitalist countries, such as the UK (Study 1) and the US (Studies 2 and 3). An increasing body of empirical evidence indicates that ideological variables can show different relations with psychological correlates in the post‐Communist context than in traditionally Capitalist societies (e.g. Cichocka, Bilewicz, Jost, Marrouch, & Witkowska, 2016; Cichocka & Jost, 2014; Kossowska & Van Hiel, 2003; Malka, Soto, Inzlicht, & Lelkes, 2014). Therefore, we conducted a survey in a post–Communist country (Poland) and tested whether our findings would replicate in this context.

Moreover, in Study 4, we added several other control variables: the Big Five personality traits (McCrae & Costa, 1987) and collective narcissism (Golec de Zavala et al., 2009). Because several basic personality traits (such as low Agreeableness) are associated with narcissism (Holtzman, Vazire, & Mehl, 2010; O'Boyle, Forsyth, Banks, Story, & White, 2015; Paulhus & Williams, 2002; Stronge, Cichocka, & Sibley, 2016) as well as ideological attitudes and prejudice (e.g. Ekehammar et al., 2004; Sibley & Duckitt, 2008; Turner, Dhont, Hewstone, Prestwich, & Vonofakou, 2014; Van Hiel, Cornelis, & Roets, 2007), we tested if the observed associations between our key variables still hold after accounting for the effects of the Big Five personality traits (McCrae & Costa, 1987).

We also accounted for the effects of collective narcissism—an unrealistically positive image of the in‐group which requires external validation (Golec de Zavala et al., 2009). Collective narcissism is a robust predictor of prejudice (for a review see Cichocka, 2016). Because collective narcissism tends to positively correlate with individual narcissism, it is important to examine the unique associations these variables have with outgroup attitudes. In three studies, Golec de Zavala, Cichocka, and Iskra‐Golec (2013) manipulated in‐group threat to investigate its impact on outgroup hostility, and measured individual and collective narcissism as well as SDO before the manipulation. They found that individual narcissism was correlated both with SDO and outgroup hostility, yet in these experimental studies, the effect of individual narcissism on outgroup hostility became non‐significant after adjusting for collective narcissism. In another study, individual narcissism was associated with interpersonal aggressiveness, but not with negative racial attitudes, although the latter were predicted by collective narcissism (Golec de Zavala et al., 2009). Therefore, in Study 4, we tested the associations of narcissism with RWA (free of SDO), SDO (free of RWA), and prejudice while controlling for national collective narcissism. In this way we aimed to rule out the possibility that the relations observed in Studies 1–3 can be explained by collective, rather than individual, narcissism. Given that Study 4 included several control variables (i.e. possible confounds) and was conducted in a very different socio‐political context, we considered it to be a conservative test of our hypotheses.

## Method

### Participants

We used data from an online survey of 926 Polish adults conducted by the Center for Research on Prejudice at the University of Warsaw. Because we analysed attitudes towards other nationalities, we excluded data from participants who reported their nationality as other than Polish (or failed to respond to the question about nationality). We also excluded 13 participants who failed to complete two or more scales measuring the five key constructs (i.e. narcissism, self‐esteem, RWA, SDO, and prejudice). For the main correlational and path analyses reported below, we used the FIML procedure in Mplus to deal with the remaining missing values. The final sample included 775 participants, 149 men and 612 women (remaining missing), aged 17–62 (*M* = 25.10, *SD* = 5.28). Participants reported their education as years of completed education (*M* = 15.91, *SD* = 2.34) and their household economic status on a scale of 1 = *bad* to 5 = *good* (*M* = 4.05, *SD* = 0.93). With this sample size, we achieved a power of .98 to detect a small effect for narcissism as predictor. The study was also well powered to detect small indirect effects.

### Measures

Narcissism was measured with the Single Item Narcissism Scale (Konrath, Meier, Bushman, & Jelte, 2014). Participants were asked to respond to the following item: ‘To what extent do you agree with this statement: I am a narcissist. (Note: The word ‘narcissist’ means egotistical, self‐focused, and vain.)’ on a scale from 1 = *this is definitely not true about me* to 7 = *this is definitely true about me*, *M =* 2.74, *SD =* 1.62.

Self‐esteem was measured with the Single Item Self‐Esteem Scale (Robins, Hendin, & Trzesniewski, 2001). 12The study also included Rosenberg's (1965) self‐esteem scale, and results are similar if this measure is used in the analyses (see Online Supplement 1, Table S6, https://osf.io/xscvw/). However, to maintain similarity with the narcissism measure, we report results for the single item in the main text. Participants were asked to respond to the item: ‘I have high self‐esteem’ on a scale from 1 = *this is definitely not true about me* to 7 = *this is definitely true about me*, *M =* 3.97, *SD =* 1.70.

Collective narcissism was measured with respect to the national group with a five item measure (Golec de Zavala, Cichocka, & Bilewicz, 2013). Participants were asked to respond to items such as ‘Poles deserve special treatment’ on a scale from 1 = *definitely disagree* to 7 = *definitely agree*, α = .87, *M =* 2.69, *SD =* 1.28.

Personality traits were measured with the Ten Item Personality Inventory (TIPI; Gosling, Rentfrow, & Swann, 2003). Participants completed pairs of items measuring extraversion, α = .66, *r* = .50, *M =* 4.44, *SD =* 1.62, agreeableness, α = .32, *r* = .20, *M =* 4.80, *SD =* 1.26, conscientiousness, α = .71, *r* = .56, *M =* 4.74, *SD =* 1.64, emotional stability, α = .56, *r* = .39, *M =* 3.70, *SD =* 1.56, and openness to experiences, α = .49, *r* = .34, *M =* 5.19, *SD =* 1.33, on a scale from 1 = *definitely disagree* to 7 = *definitely agree*.

Right‐wing authoritarianism was measured with six items based on Funke's (2005) scale, e.g. ‘Obedience and respect for authority are the most important virtues children should learn’, 1 = *definitely disagree* to 7 = *definitely agree*, α = .85, *M* = 3.46, *SD* = 1.47.

Social dominance orientation was measured with five items based on the SDO_6_ scale (see Sidanius & Pratto, 1999), e.g. ‘It's probably a good thing that certain groups are at the top and other groups are at the bottom’, 1 = *definitely disagree* to 7 = *definitely agree*, α = .81, *M* = 2.76, *SD* = 1.32.

Outgroup prejudice was measured with a measure of social distance (based on Bogardus, 1933). Participants indicated to what extent they would like to accept members of five national outgroups (Jews, Russians, Vietnamese, Gypsy, or Germans) as co‐workers, neighbours, or family members by marriage on a scale from 1 = *I would definitely oppose* to 4 = *I would definitely accept* (reverse scored), α = .93, *M* = 1.47, *SD* = 0.52. 13The survey also included measures of sexism and homophobia, and prejudice towards the poor which were part of different projects and were not analysed as our focus was on ethnic and national attitudes.


## Results

We calculated the zero‐order correlations and conducted path analyses to test our hypotheses in Mplus. We relied on FIML to deal with missing values and retain the sample of 775 respondents for all analyses. Given the limited number of items for several of the scales (e.g. narcissism, self‐esteem, personality traits), the observed scale scores were used for all analyses instead of latent factors.

Narcissism was significantly negatively correlated with RWA but was not significantly related to SDO and prejudice (Table 6). Self‐esteem showed a significant negative correlation with prejudice, but showed no significant association with RWA and SDO. Both RWA and SDO were significantly positively correlated with prejudice. Furthermore, several of the control variables showed significant associations with our key variables.

**Table 6:** Zero‐order correlations between variables (Study 4)

Measure	1	2	3	4	5	6	7	8	9	10
1. Narcissism	−									
2. Self‐esteem	.11**	−								
3. SDO	.04	.07+	−							
4. RWA	−.14***	−.03	.36***	−						
5. Collective narcissism	−.05	.06	.23***	.51***	−					
6. Extraversion	.04	.32***	−.05	−.06	.08*	−				
7. Agreeableness	−.27***	.05	−.02	.11**	.11**	.08*	−			
8. Conscientiousness	−.16***	.20***	.06+	.20***	.10**	.07+	.17***	−		
9. Emotional stability	−.14***	.41***	.09*	.10**	.08*	.08*	.27***	.26***	−	
10. Openness to experience	.02	.40***	−.01	−.10*	.05	.45***	.12***	.12***	.21***	
11. Prejudice	−.05	−.13***	.34***	.29***	.23***	−.09*	−.06	.03	−.03	−.17***

+ *p* < .10.

* *p* < .05.

** *p* < .01.

*** *p* < .001.

Path analyses were conducted to test the unique relations of narcissistic and non‐narcissistic self‐evaluations with SDO (free of RWA) and RWA (free of SDO) as well as the indirect relations with prejudice. As in Studies 2 and 3, we first tested paths from narcissistic and non‐narcissistic self‐evaluations to SDO after controlling for RWA, and then the paths to RWA after controlling for SDO, respectively. Furthermore, these models included paths from RWA and SDO to prejudice and also the direct paths from narcissistic and non‐narcissistic self‐evaluations to prejudice.

**Figure 3:**
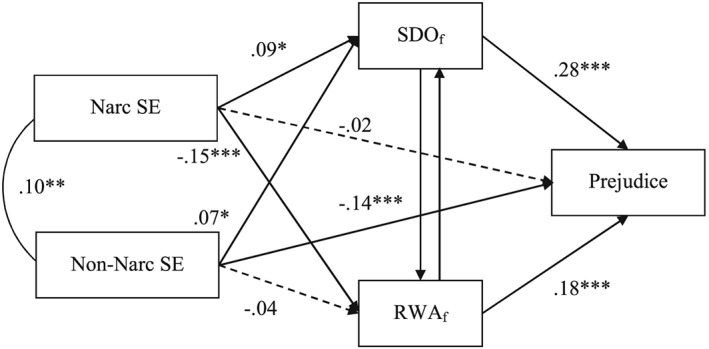
Model results of Study 4 showing the effects (standardized estimates) of narcissistic and non‐narcissistic self‐evaluation on prejudice via SDO (free of RWA) and RWA (free of SDO). *Note.* Narc SE = Narcissistic self‐evaluation; Non‐narc SE = Non‐narcissistic self‐evaluation. The results were obtained from two separate models in order to estimate the associations for SDO_f_ = SDO accounting for RWA (i.e. including the path from RWA to SDO) and for RWA_f_ = RWA accounting for SDO (i.e. including the path from SDO to RWA). * *p* < .05. ** *p* < .01. *** *p* < .001. Dashed arrows represent non‐significant paths.

Replicating the findings of Studies 1–3, the results of these analyses (Table 2, last columns) demonstrated that narcissistic self‐evaluation was significantly positively related to SDO (free of RWA) and significantly negatively related to RWA (free of SDO), confirming Hypotheses 1 and 2 (Figure 3). As expected, RWA (free of SDO) and SDO (free of RWA) were, in turn, significantly positively related to prejudice. Furthermore, non‐narcissistic self‐evaluation showed a significant, negative association with prejudice, whereas the direct path from narcissistic self‐evaluations to prejudice was non‐significant. Noteworthy, the associations for non‐narcissistic self‐evaluation were different in this Polish sample as compared to the associations observed in the UK and US samples. Non‐narcissistic self‐evaluation was significantly positively related to SDO (free of RWA) and not significantly related to RWA (free of SDO).

The analyses also confirmed Hypotheses 3 and 4, showing a positive indirect association between narcissistic self‐evaluation and prejudice via SDO (free of RWA), indirect effect = .008 [.002, .016] (standardized estimate = .024), and a negative, indirect association between narcissistic self‐evaluation and prejudice through RWA (free of SDO), indirect effect = −.009 [−.016, −.004] (standardized estimate = −.027). The indirect associations of non‐narcissistic self‐evaluation and prejudice through SDO (free of RWA) and through RWA (free of SDO) were not significant, indirect effects = .006 [.000, .013] (standardized estimate = .019), and −.002 [−.007, .002] (standardized estimate = −.006), respectively.

Importantly, conducting the same analyses but additionally including collective narcissism, Big Five personality traits, and gender as statistical controls yielded a similar pattern of results. The associations between narcissistic self‐evaluation and SDO (free of RWA) and between narcissistic self‐evaluation and RWA (free of SDO) were somewhat weaker as compared to the results without controlling for Big Five personality traits and collective narcissism, but remained significant (see Table 7). 14The results remained similar when we further controlled for education and economic status (see https://osf.io/xscvw/). Also, the indirect effects of narcissistic self‐evaluation and prejudice were similar, although less pronounced, with the indirect effect via SDO (free of RWA) being non‐significant, indirect effect = .006 [.000, .014] (standardized estimate = .019), and the indirect effect via RWA (free of SDO) being significant, indirect effect = −.003 [−.008, −.001] (standardized estimate = −.010).

**Table 7:** Results (standardized estimates) of model tests investigating the associations of narcissistic and non‐narcissistic self‐evaluations with SDO (free of RWA), RWA (free of SDO), and ethnic prejudice, controlling for collective narcissism, big five personality traits, and gender (Study 4)

	SDO_f_		RWA_f_		Ethnic prejudice	
	*β* [95% CIs]	*p*	*β* [95% CIs]	*p*	*β* [95% CIs]	*p*
Narcissistic SE	.071 [.003, .140]	.042	−.086 [−.146, −.027]	.005	−.040 [−.108, .029]	.260
Non‐Narc SE	.042 [−.037, .121]	.303	−.052 [−.121, .017]	.137	−.108 [−.186, −.029]	.007
Collective Narcissism	.066 [−.009, .141]	.087	.434 [.381, .488]	<.001	.119 [.045, .194]	.002
Extraversion	−.045 [−.119, .030]	.241	−.023 [−.088, .042]	.488	.019 [−.055, .093]	.618
Agreeableness	−.050 [−.119, .020]	.164	.037 [−.024, .098]	.235	−.071 [−.141, −.002]	.044
Conscientiousness	.005 [−.065, .075]	.894	.136 [.076, .196]	<.001	.021 [−.048, .091]	.544
Emotional Stability	.022 [−.055, .099]	.573	.025 [−.042, .092]	.462	.005 [−.071, .082]	.891
Openness	.016 [−.060, .091]	.687	−.108 [−.174, −.042]	.001	−.127 [−.203, −.051]	.001
Gender (1 = M, 0 = F)	.130 [.062, .199]	<.001	.017 [−.043, .078]	.571	.003 [−.065, .072]	.924
SDO_f_					.273 [.205, .341]	<.001
RWA_f_					.113 [.033, .193]	.006

*Note:* Narcissistic SE = Narcissistic self‐evaluation; Non‐narc SE = Non‐narcissistic self‐evaluation. The paths to SDO_f_ and RWA_f_ were calculated in two separate models which allowed to estimate the associations for SDO, accounting for RWA (i.e. by including the path from RWA to SDO) and for RWA, accounting for SDO (i.e. by including the path from SDO to RWA).

Overall, Study 4 confirmed our predictions in a different socio‐political context (post‐Communist Poland) and showed that the pattern of associations held even after accounting for a range of personality characteristic as well as collective narcissism. Narcissistic self‐evaluation did not have a significant total effect on prejudice, but indirect effect analyses demonstrated that it was both negatively associated with prejudice via RWA (free of SDO) and positively associated with prejudice with SDO (free of RWA), although the latter effects were relatively weaker and non‐significant after we introduced controls in the model. Overall, although largely consistent with the results of Studies 1–3, the effects observed in Study 4 were small, which could be due to the use of single‐item measures of self‐evaluation.

## General Discussion

In a series of four studies, conducted in three different socio‐political contexts (i.e. the UK, the USA, and Poland), we investigated the associations between individual self‐evaluations, different social‐attitudinal dimensions of ideology (that is SDO and RWA; Studies, 1–4), and ethnic prejudice (Studies 2–4). Unlike most previous studies investigating these relations (Hodson et al., 2009; Jost et al., 2003; Onraet, Van Hiel, & Dhont, 2013), we distinguished between narcissistic and non‐narcissistic self‐evaluations to test their unique relations with right‐wing ideological beliefs and prejudice. Furthermore, we also statistically accounted for any overlap between SDO and RWA. Implementing this strategy assured we examined the unique associations between narcissistic self‐evaluation and the two components of ideological attitudes. Importantly, we used reliable scales which were not too strongly correlated (all correlations between the four key variables were below .40), which suggests that there should be enough variance to analyse partial scores (Lynam, Hoyle, & Newman, 2006). By using this approach, we demonstrated, for the first time, that narcissistic self‐evaluations show different relations with SDO (free of RWA) and RWA (free of SDO), which in turn are positively related to ethnic prejudice.

### Narcissism, right‐wing ideologies, and prejudice

Confirming Hypothesis 1, narcissistic self‐evaluation was associated with higher levels of SDO (free of RWA), and thus expressed greater support for group‐based dominance and social inequality (Pratto et al., 1994). This finding is consistent with the idea that narcissism, even after controlling for self‐esteem, is associated with a stronger need to establish power over others and to satisfy the inflated feelings of entitlement and superiority. Through the positive association of narcissistic self‐evaluation and SDO (free of RWA), narcissism was further indirectly linked to greater ethnic prejudice, supporting Hypothesis 3. Displaying greater prejudice towards ethnic minority groups can be considered one way of how narcissists express their superiority strivings. These findings thus suggest that narcissists would hold greater prejudicial attitudes towards ethnic outgroups to the extent that these prejudices satisfy their feelings of entitlement, and meet their desires to dominate and exploit others. Yet, it seems rather unlikely that narcissistic individuals will express support for any social hierarchy irrespective of their own (envisioned) position in that hierarchy. Indeed, recent research has shown that narcissists are more likely to endorse social hierarchy and inequality as long as these hierarchies have a self‐serving function and help them be ‘on top’. However, because of their personal dominance strivings, narcissists are less likely to support social hierarchies in which they cannot achieve a high position (Zitek & Jordan, 2016).

In line with Hypothesis 2, a negative relation emerged between narcissistic self‐evaluation and RWA (free of SDO). This finding is consistent with the idea that narcissistic self‐evaluation captures feelings of uniqueness and grandiosity, and is linked to nonconformity (e.g. Goncalo et al., 2010). Indeed, RWA (free of SDO) encompasses the ideological belief that people should conform to the social norms and traditions endorsed by the society (e.g. Bilewicz, Soral, Marchlewska, & Winiewski, 2015). These ideological beliefs go against narcissists' individualistic desires to be independent and rebellious. Through the negative association with RWA (free of SDO), narcissistic self‐evaluation was further indirectly linked to lesser ethnic prejudice, supporting Hypothesis 4. Narcissists should hold less prejudicial attitudes towards ethnic outgroups to the extent that these target outgroups do not conform to societal traditions and norms. In other words, the negative indirect association between narcissistic self‐evaluation and prejudice may reflect narcissistic tendencies to view themselves as being open to novelty and resisting traditional in‐group norms.

Taken together, the opposite relations between narcissistic self‐evaluation and SDO versus RWA (once their overlap is accounted for) shed new light on the link between narcissism and ethnic prejudice. At the zero‐order correlational level, narcissism did not show a clear relation with prejudice: these variables were positively correlated in Study 2 but were not significantly correlated in Studies 3 and 4. However, due to the differential effects via SDO (free of RWA) and RWA (free of SDO), the results showed that narcissistic self‐evaluation was indirectly linked to greater prejudice through higher SDO but to lesser prejudice through lower RWA. Study 4 demonstrated that these indirect effects of narcissism on prejudice were similar, albeit admittedly weaker, after accounting for the effects of personality traits and collective narcissism—the inflated image of the *in‐group*, which has previously been linked to negative outgroup attitudes (e.g. Golec de Zavala et al., 2009; Golec de Zavala, Cichocka, & Iskra‐Golec, 2013).

Furthermore, the differential relations of narcissistic self‐evaluation with SDO (free of RWA) and RWA (free of SDO) emerged even when we additionally controlled for other personality characteristics such as psychopathy and Machiavellianism (Paulhus & Williams, 2002; Study 3). Specifically, both psychopathy and Machiavellianism were positively correlated with SDO, and the inclusion of these variables in the model tended to weaken the association between narcissistic self‐evaluation and SDO (free of RWA). Yet, the latter association remained significant and therefore cannot fully be explained by psychopathy and Machiavellianism. The association between narcissistic self‐evaluation and RWA (free of SDO) was also weaker (and non‐significant) after the inclusion of psychopathy and Machiavellianism, even though the latter variables did not show a significant relation with RWA. Furthermore, accounting for the Big Five personality traits (McCrae & Costa, 1987; Study 4) did not meaningfully affect the observed relations between narcissistic self‐evaluation, SDO (free of RWA) and RWA (free of SDO), and prejudice.

Despite the differences in the strength of the effects between studies, we obtained a fairly consistent pattern of results across different political contexts: two traditionally Capitalist countries (the US and the UK) as well as a post‐Communist country (Poland). These countries also differ with respect to levels of ethnic diversity and their history of ethnic relations. Notwithstanding the diverse cultural and political backgrounds, we found very similar patterns of results, suggesting that narcissists might be attracted to a similar set of ideological and intergroup beliefs across countries. Future research would do well to validate these findings beyond the context of individualistic cultures, which tend to show relatively high levels of narcissism (Foster, Campbell, & Twenge, 2003).

Future work should also examine links between narcissism and prejudicial attitudes towards a greater variety of outgroups. While our research focused on ethnic prejudice, future work may identify specific groups that are particularly likely to evoke prejudicial attitudes among narcissists. Duckitt and Sibley (2007; see also Asbrock, Sibley, & Duckitt, 2010) demonstrated that SDO predicted prejudice towards groups that are socially subordinate, and therefore derogated in society (e.g. unemployed people, psychiatric patients), while RWA predicted prejudice towards groups that are perceived as threatening to the social order (e.g. drug dealers, gang members). Combining these previous findings with our findings showing that narcissistic self‐evaluation is positively associated with SDO (free of RWA), we could expect that narcissists might be particularly prejudiced towards derogated groups. The negative link between narcissistic self‐evaluation and RWA (free of SDO), however, might indicate that narcissists are less concerned with those who threaten the norms of the socio‐political system. Future work should examine these interesting possibilities.

### Non‐narcissistic self‐esteem and right‐wing ideologies

Our hypotheses focused on the correlates of narcissism. To test the unique associations of narcissistic self‐evaluation with ideological attitudes and prejudice, we also included self‐esteem in the analyses. In this way, we accounted for the variance narcissism and self‐esteem share (Cichocka, Marchlewska, et al., 2016; Marchlewska & Cichocka, 2017; Paulhus et al., 2004). We did not formulate specific hypotheses regarding the associations between non‐narcissistic self‐evaluation and right‐wing attitudes given the weak and mixed effects obtained in previous work (see Jost et al., 2003; Onraet, Van Hiel, & Dhont, 2013). Nevertheless, our findings offer some new insights into the nature of these relations.

One interesting finding was that non‐narcissistic self‐evaluation was positively associated with RWA (free of SDO), as observed in Studies 1–3 (conducted in the UK, and the USA), but not in Study 4 (conducted in Poland). This result is somewhat surprising given that past work linked non‐narcissistic self‐evaluation to lower anti‐social tendencies and interpersonal aggressiveness (Locke, 2009; Paulhus et al., 2004), both of which would suggest a positive association with authoritarian aggression. By accounting for the variance shared with SDO, we might have been better suited to demonstrate the positive link between RWA and non‐narcissistic self‐evaluation. Indeed, some previous studies have shown that endorsement of right‐wing beliefs is linked to higher levels of self‐esteem (e.g. Van Hiel & Brebels, 2011) and other explicit indicators of well‐being and life satisfaction (MacInnis, Busseri, Choma, & Hodson, 2013; Napier & Jost, 2008; Onraet, Van Assche, Roets, Haesevoets, & Van Hiel, 2017). According to system‐justification theory (Jost & Banaji, 1994), endorsement of right‐wing beliefs is a system‐justifying ideology associated with legitimization of the societal status‐quo and with greater perceptions of fairness of the current social, political, and economic system. This serves a palliative function, which can explain the higher levels of self‐reported happiness among conservatives than among liberals (Napier & Jost, 2008). This happiness gap has been shown to be context dependent, with stronger associations between right‐wing attitudes and well‐being in more threatening contexts or contexts with higher levels of inequality (see Onraet et al., 2017; Napier & Jost, 2008). Contextual differences could be one explanation for why we found the positive association between non‐narcissistic self‐evaluation and RWA (free of SDO) in the British and American samples, but not in the Polish sample. It is, however, unclear what contextual factors may have caused the difference in the current set of studies and future studies could further investigate this issue.

Another possibility is that responses to our explicit measures of self‐evaluation were affected by social desirability. Recent work suggests that although right‐wingers indeed declare greater happiness, this effect can be attributed to self‐enhancement tendencies (Wojcik, Hovasapian, Graham, Motyl, & Ditto, 2015). In fact, Wojcik et al. (2015) showed that right‐wingers were *less* likely to display happiness‐related behaviour than liberals. Although self‐enhancement strategies should be primarily linked to narcissism, our operationalization of non‐narcissistic self‐evaluation still relied on self‐report measures and might have captured cultural differences in expressing positive or negative judgements about oneself. For example, research suggests that there is a norm of expressing more self‐negativity in Poland as compared to Western countries (such as the US; Wojciszke, 2005). If Poles are less likely to show self‐enhancement while responding to measures of explicit self‐esteem, this might explain why non‐narcissistic self‐evaluation was not linked to right‐wing ideologies in this country.

Non‐narcissistic self‐evaluation did not show such positive associations with SDO (free of RWA). On the contrary, in Studies 1, 2, and 3, non‐narcissistic self‐evaluation was negatively linked to SDO (free of RWA). This is consistent with Sniderman and Citrin's (1971) assertion that low feelings of self‐worth might be linked to right‐wing ideology that tolerates seeing others as weaker and less competent and, thus, helps the individual compensate for low self‐esteem. Self‐esteem was also found to be weakly, negatively related to SDO in the meta‐analysis by Onraet, Van Hiel, and Dhont (2013). Nevertheless, in our studies, this pattern of results was only present once we accounted for narcissism and RWA. These results may explain why previous studies and meta‐analytic reviews (Jost et al., 2003; Onraet et al., 2013) did not obtain convincing evidence in support of the classic hypothesis predicting a negative link between self‐esteem and right‐wing ideology (Adorno et al., 1950; Sniderman & Citrin, 1971; Wilson, 1973). Moreover, in Study 4, non‐narcissistic self‐evaluation was positively, albeit weakly, linked to SDO (free of RWA). Thus, our findings particularly emphasize the more consistent role of higher narcissistic self‐evaluation, rather than lower non‐narcissistic self‐evaluation, in predicting SDO across various contexts.

### Implications

Overall, our findings contribute to the understanding of the psychological underpinnings of ideological convictions. They extend past theorizing on the needs and motives associated with right‐wing ideologies. Our findings corroborate the motivated social cognition model of ideology (Jost et al., 2003; Jost, Federico, & Napier, 2009) by demonstrating that feelings of self‐worth are associated with right‐wing political inclinations. They also extend the dual process model of ideology and prejudice (Duckitt, 2001; Duckitt & Sibley, 2010) by showing that RWA and SDO are differentially associated with self‐evaluations. Past work conducted within this framework demonstrated that RWA and SDO were associated with different personality traits (e.g. Sibley & Duckitt, 2008). The results of Study 4 demonstrated differential relations of RWA (free of SDO) and SDO (free of RWA) with narcissistic self‐evaluations, over and above their links with the Big Five.

Our research also shows the utility of partialling for uncovering clearer links between variables; yet, it also highlights the need for a careful approach to controlling for overlapping constructs and interpreting the findings. For example, we demonstrated that certain effects are better observed once the variance shared between SDO and RWA is accounted for. However, the partialled nature of these variables needs to be acknowledged in any interpretation of the results. We hope that future work looking at different dimensions of ideology and self‐evaluation will pay greater attention to the implementation and interpretation of co‐varying shared variance.

### Limitations and future directions

Relying on partialled variables comes with certain challenges to reliability and interpretability (see Lynam et al., 2006). Therefore, we clarified the conceptual definitions of the partialled measures so that the findings can be interpreted as meaningfully and accurately as possible. We also used reliable measures that showed only moderate inter‐correlations, and demonstrated the effects with structural equation modelling. Nevertheless, partialling makes real‐life applications more challenging. For example, narcissism and self‐esteem often co‐vary, and, therefore, it might be difficult to identify individuals with high narcissism but low self‐esteem (for a similar discussion see Stoeber, Kobori, & Brown, 2014 in the context of perfectionism, and see Cichocka, 2016; Cichocka et al., in press in the context of narcissistic and non‐narcissistic in‐group positivity). Thus, future studies should aim to develop tools that would capture the concepts more directly, without the need to co‐vary their shared variance.

One way to observe the associations more clearly could be to distinguish between different dimensions of narcissism (for a review, see Krizan & Herlache, in press). Ackerman et al. (2011) identified three subscales of the NPI: Leadership/Authority, Grandiose exhibitionism and Entitlement/Exploitativeness. It could be argued that the components of Leadership/Authority and Entitlement/Exploitativeness best capture the dominance tendencies of narcissists, and therefore, these components might show the most pronounced positive relations with SDO (free of RWA). Because Grandiose exhibitionism best captures the narcissistic feelings of uniqueness, this component might be most strongly negatively associated with RWA (free of SDO). To explore these ideas, we conducted additional, exploratory analyses for Studies 1–3 using these three subscales of the NPI (see Online Supplement 1, Tables S4.1–S4.6, https://osf.io/xscvw/). These analyses demonstrated that all three subcomponents of narcissistic self‐evaluation were positively associated with SDO (free of RWA). Grandiose exhibitionism was the only subcomponent of narcissistic self‐evaluation that consistently showed a negative association with RWA (free of SDO) across the three studies, although negative associations were also observed for the Leadership/Authority component in Studies 1 and 3 and for the Exploitativeness component in Study 3. The results of these exploratory analyses should, however, be treated with caution given the poor measurement quality of the entitlement component in the NPI (Krizan & Herlache, in press; see also Online Supplement 1, Tables S4.1–S4.3 for the reliabilities of the subscales).

Future research might consider employing other measures of narcissism. For example, Back et al.'s (2013) Narcissistic Admiration and Rivalry Questionnaire distinguishes two aspects of grandiose narcissism: admiration (characterized by grandiosity, uniqueness, and charmingness), and rivalry (characterized by devaluation, supremacy, and aggressiveness). Because narcissistic admiration is linked to both entitlement and grandiosity, for this dimension we would expect a similar pattern of results as the one we obtained in the current research, especially once we adjust for self‐esteem (which tends to be positively associated with narcissistic admiration). However, due to its supremacy component, narcissistic rivalry might show a stronger link with SDO, possibly even without the need to account for self‐esteem (which tends to be negatively associated with narcissistic rivalry; see Back et al., 2013).

Recent work also points to the existence of a different dimension of narcissistic personality characterized by vulnerability (Cain, Pincus, & Ansell, 2008). Vulnerable narcissism is linked to reactivity to self‐threats. Therefore, this dimension of narcissism might be positively (rather than negatively) associated with RWA, given that RWA tends to be associated with higher threat sensitivity (for overviews see Cichocka & Dhont, in press; Onraet, Van Hiel, Dhont, & Pattyn, 2013). Because similarly to narcissistic grandiosity, narcissistic vulnerability is associated with feelings of entitlement and self‐importance (Krizan & Herlache, in press), vulnerable narcissism might also predict higher SDO. Future research should examine these possibilities empirically, especially testing whether the proposed associations would hold after adjusting for the low self‐esteem or neuroticism associated with vulnerable narcissism (see Miller et al., in press).

Finally, because the current set of studies relied on cross‐sectional designs, we cannot make causal claims. Therefore, it is important to bear in mind that the indirect effects reported here should be treated as indicators of how well different components of ideological attitudes account for the links between narcissistic self‐evaluation and prejudice, rather than as causal models. The order of variables in our model was informed by the assumption that narcissism and self‐esteem are relatively basic personality predispositions, which should predict broader ideological attitudes, which further predict intergroup attitudes. Nevertheless, it is also possible that these variables affect each other (Cunningham, Nezlek, & Banaji, 2004). Future studies could employ longitudinal methods to corroborate the estimates of the indirect effects models we proposed here.

## Conclusions

By distinguishing between narcissistic and non‐narcissistic self‐evaluations and investigating different dimensions of ideological attitudes, our studies uniquely contribute to the debate on whether individual self‐evaluations are positively or negatively related to right‐wing beliefs and prejudice. Our studies indicate that narcissistic self‐evaluations were indirectly and positively associated with prejudice through higher SDO (accounting for RWA), yet indirectly and negatively associated with prejudice through lower RWA (accounting for SDO). These findings emphasize the complex nature of the relations and might explain why previous work has provided inconsistent evidence on the associations of individual self‐evaluations with right‐wing beliefs and outgroup attitudes.

## Supporting information

Table S1. *Results (standardized estimates) of models testing the associations of* ***narcissism*** *with SDO (free of RWA) and RWA (free of SDO) (Studies1–4) and ethnic prejudice (Studies 2–4)* ***without accounting for self‐esteem***
Table S2. *Results (standardized estimates) of models testing the associations of* ***self‐esteem*** *with SDO (free of RWA) and RWA (free of SDO) (Studies1–4) and ethnic prejudice (Studies 2–4)* ***without accounting for narcissism***
Table S3. *Results (standardized estimates) of models only testing the associations of* ***narcissistic self‐evaluation and non‐narcissistic self‐evaluation with SDO (without controlling for RWA) and RWA (without controlling for SDO)*** *(Studies1–4) and ethnic prejudice (Studies 2–4)*
Table S4.1 *Descriptive statistics, scale reliabilities and zero‐order correlations between manifest variables in Study 1, including the three subcomponents of Narcissism*
Table S4.2 *Descriptive statistics, scale reliabilities and zero‐order correlations between manifest variables in Study 2, including the three subcomponents of Narcissism*
Table S4.3 *Descriptive statistics, scale reliabilities, and zero‐order correlations between manifest variables in Study 3, including the three subcomponents of Narcissism*
Table S4.4 *Results (standardized estimates) of latent models testing the associations of the* ***Leadership/Authority subcomponent*** *of narcissistic self‐evaluation and non‐narcissistic self‐evaluation with SDO (free of RWA) and RWA (free of SDO) (Studies1–3) and ethnic prejudice (Studies 2–3)*
Table S4.5 *Results (standardized estimates) of latent models testing the associations of the* ***Grandiose Exhibitionism*** *subcomponent of narcissistic self‐evaluation and non‐narcissistic self‐evaluation with SDO (free of RWA) and RWA (free of SDO) (Studies1–3) and ethnic prejudice (Studies 2–3).*
Table S4.6 *Results (standardized estimates) of latent models testing the associations of the* ***Entitlement/Exploitativeness subcomponent*** *of narcissistic self‐evaluation and non‐narcissistic self‐evaluation with SDO (free of RWA) and RWA (free of SDO) (Studies1–3) and ethnic prejudice (Studies 2–3).*
Table S5.1 *Zero‐order correlations between manifest variables separately for male and female respondents (Study 1)*
Table S5.2 *Zero‐order correlations between manifest variables separately for male and female respondents (Study 2)*
Table S5.3 *Zero‐order correlations between manifest variables, separately for male (n = 118) and female (n = 171) respondents (Study 3)*
Table 5.4 *Zero‐order correlations between variables for male respondents (n = 149) in Study 4.*
Table S5.5 *Zero‐order correlations between variables for female respondents (n = 612) in Study 4.*
Table S5.6 *Results (standardized estimates) of models* ***only including male respondents*** *testing the associations of the narcissistic self‐evaluation and non‐narcissistic self‐evaluation with SDO (free of RWA) and RWA (free of SDO) (Studies1–4) and ethnic prejudice (Studies 2–4)*
Table S5.7 *Results (standardized estimates) of models only including* ***female respondents*** *testing the associations of the narcissistic self‐evaluation and non‐narcissistic self‐evaluation with SDO (free of RWA) and RWA (free of SDO) (Studies1–4) and ethnic prejudice (Studies 2–4)*
Table S6. *Model results (standardized estimates) investigating the associations of narcissistic self‐evaluation and non‐narcissistic self‐evaluation (Rosenberg self‐esteem scale) with SDO (free of RWA) and RWA (free of SDO) and ethnic prejudice (Study 4).*

Supporting info item
