# Supplementary material for: On Self‐Love and Outgroup Hate: Opposite Effects of Narcissism on Prejudice via Social Dominance Orientation and Right‐Wing Authoritarianism
Source: Eur J Pers. 2017 Aug 4;31(4):366–84. doi: 10.1002/per.2114 (PMC5601291; doi:10.1002/per.2114)
Supplement: Supplementary file 1 — Table S1. Results (standardized estimates) of models testing the associations of narcissism with SDO (free of RWA) and RWA (free of SDO) (Studies1–4) and ethnic prejudice (Studies 2–4) without accounting for self‐esteem Table S2. Results (standardized estimates) of models testing the associations of self‐esteem with SDO (free of RWA) and RWA (free of SDO) (Studies1–4) and ethnic prejudice (Studies 2–4) without accounting for narcissism Table S3. Results (standardized estimates) of models only testing the associations of narcissistic self‐evaluation and non‐narcissistic self‐evaluation with SDO (without controlling for RWA) and RWA (without controlling for SDO) (Studies1–4) and ethnic prejudice (Studies 2–4) Table S4.1 Descriptive statistics, scale reliabilities and zero‐order correlations between manifest variables in Study 1, including the three subcomponents of Narcissism Table S4.2 Descriptive statistics, scale reliabilities and zero‐order correlations between manifest variables in Study 2, including the three subcomponents of Narcissism Table S4.3 Descriptive statistics, scale reliabilities, and zero‐order correlations between manifest variables in Study 3, including the three subcomponents of Narcissism Table S4.4 Results (standardized estimates) of latent models testing the associations of the Leadership/Authority subcomponent of narcissistic self‐evaluation and non‐narcissistic self‐evaluation with SDO (free of RWA) and RWA (free of SDO) (Studies1–3) and ethnic prejudice (Studies 2–3) Table S4.5 Results (standardized estimates) of latent models testing the associations of the Grandiose Exhibitionism subcomponent of narcissistic self‐evaluation and non‐narcissistic self‐evaluation with SDO (free of RWA) and RWA (free of SDO) (Studies1–3) and ethnic prejudice (Studies 2–3). Table S4.6 Results (standardized estimates) of latent models testing the associations of the Entitlement/Exploitativeness subcomponent of narcissistic self‐evaluation and non‐narcissistic se [file PER-31-366-s001.docx]

**Online Supplement - Cichocka, Dhont, & Makwana**

On Self-love and Outgroup Hate: Opposite Effects of Narcissism on Prejudice via Social Dominance Orientation and Right-Wing Authoritarianism

*European Journal of Personality*

**Additional analyses without partialling**

We conducted additional analyses testing the associations of narcissism (without controlling for self-esteem, Table S1), and self-esteem (without controlling for narcissism, Table S2) with SDO (free of RWA) and RWA (free of SDO). We also report the associations between narcissistic and non-narcissistic self-evaluation with RWA and SDO (without controlling for the shared variance between RWA and SDO, Table S3).

Table S1

*Results (standardized estimates) of models testing the associations of* ***narcissism*** *with SDO (free of RWA) and RWA (free of SDO) (Studies1-4) and ethnic prejudice (Studies 2-4)* ***without accounting for self-esteem***

|  | Study 1 | | Study 2 | | Study 3 | | Study 4 | |
| --- | --- | --- | --- | --- | --- | --- | --- | --- |
| Associations | *β* | *p* | *β* | *p* | *β* | *p* | *β* | *p* |
| Narcissism 🡪 SDO_f_ | .226 | < .001 | .361 | < .001 | .406 | < .001 | .093 | .006 |
| Narcissism 🡪 RWA_f_ | -.187 | .001 | -.071 | .177 | -.194 | .008 | -.154 | < .001 |
| Narcissism 🡪 prejudice | / | / | .019 | .649 | -.095 | .123 | -.032 | .349 |
| SDO_f_ 🡪 Prejudice | / | / | .497 | < .001 | .717 | < .001 | .271 | < .001 |
| RWA_f_ 🡪 Prejudice | / | / | .342 | < .001 | .138 | .023 | .186 | < .001 |

*Note*. The paths to SDO_f_ and RWA_f_ were calculated in two separate models which allowed us to estimate the associations for SDO, accounting for RWA (i.e. by including the path from RWA to SDO) and for RWA, accounting for SDO (i.e., by including the path from SDO to RWA).

Table S2

*Results (standardized estimates) of models testing the associations of* ***self-esteem*** *with SDO (free of RWA) and RWA (free of SDO) (Studies1-4) and ethnic prejudice (Studies 2-4)* ***without accounting for narcissism***

|  | Study 1 | | Study 2 | | Study 3 | | Study 4 | |
| --- | --- | --- | --- | --- | --- | --- | --- | --- |
| Associations | *β* | *p* | *β* | *p* | *β* | *p* | *β* | *p* |
| Self-esteem 🡪 SDO_f_ | .011 | .824 | -.026 | .598 | -.160 | .007 | .075 | .024 |
| Self-esteem 🡪 RWA_f_ | .090 | .143 | .181 | < .001 | .250 | <.001 | -.051 | .125 |
| Self-esteem 🡪 prejudice | / | / | .066 | .085 | -.078 | .112 | -.142 | .809 |
| SDO_f_ 🡪 Prejudice | / | / | .506 | < .001 | .661 | < .001 | .278 | < .001 |
| RWA_f_ 🡪 Prejudice | / | / | .328 | < .001 | .179 | .003 | .184 | < .001 |

*Note*. The paths to SDO_f_ and RWA_f_ were calculated in two separate models which allowed us to estimate the associations for SDO, accounting for RWA (i.e. by including the path from RWA to SDO) and for RWA, accounting for SDO (i.e., by including the path from SDO to RWA).

Table S3

*Results (standardized estimates) of models only testing the associations of* ***narcissistic self-evaluation and non-narcissistic self-evaluation with SDO (without controlling for RWA) and RWA (without controlling for SDO)*** *(Studies1-4) and ethnic prejudice (Studies 2-4)*

|  | Study 1 | | Study 2 | | Study 3 | | Study 4 | |
| --- | --- | --- | --- | --- | --- | --- | --- | --- |
| Associations | *β* | *p* | *β* | *p* | *β* | *p* | *β* | *p* |
| Narcissism – Self-Esteem | .426 | < .001 | .296 | < .001 | .190 | .003 | .104 | .004 |
| Narc SE 🡪 SDO | .186 | .003 | .401 | < .001 | .428 | < .001 | .034 | .342 |
| Non-narc SE 🡪 SDO | -.017 | .772 | -.085 | .085 | -.151 | .004 | .062 | .087 |
| Narc SE 🡪 RWA | -.183 | .004 | -.002 | .969 | -.063 | .391 | -.137 | < .001 |
| Non-narc SE 🡪 RWA | .195 | .008 | .192 | .001 | .237 | .001 | -.013 | .715 |
| Narc SE 🡪 prejudice | / | / | -.004 | .940 | -.076 | .254 | -.018 | .590 |
| Non-narc SE 🡪 Prejudice | / | / | .067 | .115 | -.057 | .282 | -.141 | < .001 |
| SDO 🡪 Prejudice | / | / | .508 | < .001 | .699 | <.001 | .280 | < .001 |
| RWA 🡪 Prejudice | / | / | .327 | < .001 | .158 | .013 | .180 | < .001 |

*Note*. Narc SE = Narcissistic Self-Evaluation; Non-narc SE = Non-narcissistic Self-Evaluation.

**Analyses for narcissism subscales**

We also conducted exploratory analyses for Studies 1-3 using the three subscales of the NPI identified by Ackerman and colleagues (2013): Leadership/Authority, Grandiose exhibitionism and Entitlement/Exploitativeness. These are reported in Tables S4.1 – S4.6. Note that the internal consistency reliability of the Entitlement/Exploitativeness subscale was below acceptable standards for Studies 1 and 2. The results of the separate analyses using this subscale should therefore be interpreted with care.

Table S4.1

*Descriptive statistics, scale reliabilities and zero-order correlations between manifest variables in Study 1, including the three subcomponents of Narcissism*

| Measure | *M* | *SD* | *α* | 2. | 3. | 4. | 5. | 6. | 7. |
| --- | --- | --- | --- | --- | --- | --- | --- | --- | --- |
| 1. Narcissism | .29 | .16 | .85 | .86^***^ | .47^***^ | .74^***^ | .39^***^ | -.07 | .16^***^ |
| 2. Authority | .32 | .24 | .77 | - | .30^***^ | .49^***^ | .37^***^ | -.06 | .11^*^ |
| 3. Exhibitionism | .25 | .22 | .71 |  | - | .23^***^ | .35^***^ | -.11^*^ | .15^**^ |
| 4. Exploitativeness | .22 | .24 | .38 |  |  | - | -.05 | -.003 | .21^***^ |
| 5. Self Esteem | 4.52 | 1.21 | .91 |  |  |  | - | .12^**^ | .07 |
| 6. RWA | 3.47 | .84 | .78 |  |  |  |  | - | .38^***^ |
| 7. SDO | 2.33 | .98 | .92 |  |  |  |  |  | - |

* *p* < .05. ** *p* < .01. *** *p* < .001.

Table S4.2

*Descriptive statistics, scale reliabilities and zero-order correlations between manifest variables in Study 2, including the three subcomponents of Narcissism*

| Measure | *M* | *SD* | *α* | 2. | 3. | 4. | 5. | 6. | 7. | 8. |
| --- | --- | --- | --- | --- | --- | --- | --- | --- | --- | --- |
| 1. Narcissism | .30 | .19 | .88 | .86^***^ | .78^***^ | .51^***^ | .26^***^ | .05 | .35^***^ | .22^***^ |
| 2. Authority | .36 | .28 | .82 | - | .51^***^ | .31^***^ | .32^***^ | .08 | .26^***^ | .19^***^ |
| 3. Exhibitionism | .22 | .23 | .78 |  | - | .32^***^ | .14^**^ | -.01 | .26^***^ | .13^**^ |
| 4. Exploitativeness | .21 | .25 | .45 |  |  | - | -.17^***^ | -.05 | .26^***^ | .17^***^ |
| 5. Self Esteem | 3.76 | .92 | .93 |  |  |  | - | .18^***^ | .03 | .11^*^ |
| 6. RWA | 3.66 | 1.20 | .88 |  |  |  |  | - | .29^***^ | .44^***^ |
| 7. SDO | 2.45 | 1.26 | .95 |  |  |  |  |  | - | .60^***^ |
| 8. Prejudice | 3.18 | 1.62 | .85 |  |  |  |  |  |  | - |

* *p* < .05. ** *p* < .01. *** *p* < .001.

Table S4.3

*Descriptive statistics, scale reliabilities, and zero-order correlations between manifest variables in Study 3, including the three subcomponents of Narcissism*

| Measure | *M* | *SD* | *α* | 2. | 3. | 4. | 5. | 6. | 7. | 8. | 9. | 10. |
| --- | --- | --- | --- | --- | --- | --- | --- | --- | --- | --- | --- | --- |
| 1. Narcissism | 3.68 | 1.01 | .96 | .93^***^ | .82^***^ | .80^***^ | .45^***^ | .44^***^ | .18^**^ | -.01 | .37^***^ | .15^**^ |
| 2. Authoritativeness | 4.04 | 1.29 | .92 | - | .62^***^ | .72^***^ | .36^***^ | .37^***^ | .23^***^ | .04 | .33^***^ | .11 |
| 3. Exhibitionism | 2.91 | 1.18 | .91 |  | - | .60^***^ | .43^***^ | .33^***^ | .05 | -.08 | .32^***^ | .16^**^ |
| 4. Exploitativeness | 3.51 | 1.25 | .75 |  |  | - | .52^***^ | .52^***^ | -.05 | -.01 | .39^***^ | .17^**^ |
| 5. Psychopathy | 2.35 | 1.02 | .83 |  |  |  | - | .54^***^ | -.36^***^ | -.16^**^ | .35^***^ | .34^***^ |
| 6. Machiavellianism | 4.03 | 1.18 | .87 |  |  |  |  | - | -.22^***^ | -.07 | .34^***^ | .32^***^ |
| 7. Self Esteem | 5.32 | 1.28 | .93 |  |  |  |  |  | - | .21^***^ | -.06 | -.09 |
| 8. RWA | 3.80 | 1.37 | .92 |  |  |  |  |  |  | - | .36^***^ | .32^***^ |
| 9. SDO | 2.43 | 1.34 | .96 |  |  |  |  |  |  |  | - | .63^***^ |
| 10. Prejudice | - | - | .93 |  |  |  |  |  |  |  |  | - |

* *p* < .05, ** *p* < .01, *** *p* < .001

Table S4.4

*Results (standardized estimates) of latent models testing the associations of the* ***Leadership/Authority subcomponent*** *of narcissistic self-evaluation and non-narcissistic self-evaluation with SDO (free of RWA) and RWA (free of SDO) (Studies1-3) and ethnic prejudice (Studies 2-3)*

|  | Study 1 | | Study 2 | | Study 3 | |
| --- | --- | --- | --- | --- | --- | --- |
| Associations | *β* | *p* | *β* | *p* | *β* | *p* |
| Authority 🡪 SDO_f_ | .216 | < .001 | .331 | < .001 | .404 | < .001 |
| Non-narc SE 🡪 SDO_f_ | -.085 | .129 | -.153 | .005 | -.273 | < .001 |
| Authority 🡪 RWA_f_ | -.244 | < .001 | -.089 | .146 | -.210 | .006 |
| Non-narc SE 🡪 RWA_f_ | .191 | .006 | .214 | < .001 | .314 | < .001 |
| Authority 🡪 prejudice | / | / | .022 | .664 | -.097 | .169 |
| Non-narc SE 🡪 Prejudice | / | / | .057 | .201 | -.046 | .401 |
| SDO_f_ 🡪 Prejudice | / | / | .500 | < .001 | .702 | < .001 |
| RWA_f_ 🡪 Prejudice | / | / | .329 | < .001 | .157 | .012 |

*Note.* Non-narc SE = Non-narcissistic Self-Evaluation. The paths to SDO_f_ and RWA_f_ were calculated in two separate models which allowed us to estimate the associations for SDO, accounting for RWA (i.e. by including the path from RWA to SDO) and for RWA, accounting for SDO (i.e., by including the path from SDO to RWA).

Table S4.5

*Results (standardized estimates) of latent models testing the associations of the* ***Grandiose Exhibitionism*** *subcomponent of narcissistic self-evaluation and non-narcissistic self-evaluation with SDO (free of RWA) and RWA (free of SDO) (Studies1-3) and ethnic prejudice (Studies 2-3).*

|  | Study 1 | | Study 2 | | Study 3 | |
| --- | --- | --- | --- | --- | --- | --- |
| Associations | *β* | *p* | *β* | *p* | *β* | *p* |
| Exhibitionism 🡪 SDO_f_ | .299 | < .001 | .308 | < .001 | .413 | < .001 |
| Non-narc SE 🡪 SDO_f_ | -.123 | .036 | -.073 | .142 | -.194 | < .001 |
| Exhibitionism 🡪 RWA_f_ | -.327 | < .001 | -.118 | .042 | -.295 | < .001 |
| Non-narc SE 🡪 RWA_f_ | .225 | .001 | .198 | < .001 | .275 | < .001 |
| Exhibitionism 🡪 prejudice | / | / | -.035 | .488 | -.024 | .708 |
| Non-narc SE 🡪 Prejudice | / | / | .071 | .076 | -.074 | .143 |
| SDO_f_ 🡪 Prejudice | / | / | .517 | < .001 | .672 | < .001 |
| RWA_f_ 🡪 Prejudice | / | / | .324 | < .001 | .171 | .007 |

*Note*. Non-narc SE = Non-narcissistic Self-Evaluation. The paths to SDO_f_ and RWA_f_ were calculated in two separate models which allowed us to estimate the associations for SDO, accounting for RWA (i.e. by including the path from RWA to SDO) and for RWA, accounting for SDO (i.e., by including the path from SDO to RWA).

Table S4.6

*Results (standardized estimates) of latent models testing the associations of the* ***Entitlement / Exploitativeness subcomponent*** *of narcissistic self-evaluation and non-narcissistic self-evaluation with SDO (free of RWA) and RWA (free of SDO) (Studies1-3) and ethnic prejudice (Studies 2-3).*

|  | Study 1 | | Study 2 | | Study 3 | |
| --- | --- | --- | --- | --- | --- | --- |
| Associations | *β* | *p* | *β* | *p* | *β* | *p* |
| Exploitativeness 🡪 SDO_f_ | .332 | < .001 | .365 | < .001 | .476 | < .001 |
| Non-narc SE 🡪 SDO_f_ | .016 | .737 | .068 | .170 | -.144 | .008 |
| Exploitativeness 🡪 RWA_f_ | -.133 | .168 | -.071 | .256 | -.264 | .001 |
| Non-narc SE 🡪 RWA_f_ | .085 | .165 | .163 | .002 | .249 | < .001 |
| Exploitativeness 🡪 prejudice | / | / | .040 | .516 | -.137 | .057 |
| Non-narc SE 🡪 Prejudice | / | / | .076 | .065 | -.070 | .158 |
| SDO_f_ 🡪 Prejudice | / | / | .491 | < .001 | .737 | < .001 |
| RWA_f_ 🡪 Prejudice | / | / | .330 | < .001 | .144 | .017 |

*Note*. Non-narc SE = Non-narcissistic Self-Evaluation. The paths to SDO_f_ and RWA_f_ were calculated in two separate models which allowed us to estimate the associations for SDO, accounting for RWA (i.e. by including the path from RWA to SDO) and for RWA, accounting for SDO (i.e., by including the path from SDO to RWA). The internal consistency reliability of the Exploitativeness subscale was below acceptable standards for Studies 1 and 2. The results of the separate analyses using this subscale should therefore be interpreted with care.

**Results split by gender.**

We conducted additional analyses showing the results separately for male and female respondents. These are reported in Tables S5.1 – S5.7

Table S5.1 *Zero-order correlations between manifest variables separately for male and female respondents (Study 1)*

| Measure | Males (n = 49) | | | | | |  | Females (n = 350) | | | | | |
| --- | --- | --- | --- | --- | --- | --- | --- | --- | --- | --- | --- | --- | --- |
|  | 2 | 3 | 4 | 5 | 6 | 7 |  | 2 | 3 | 4 | 5 | 6 | 7 |
| 1. Narcissism | .89^***^ | .79^***^ | .51^***^ | .41^**^ | -.20 | .01 |  | .85^***^ | .72^***^ | .42^***^ | .41^***^ | -.04 | .16^**^ |
| 2. Authoritativeness | - | .58^***^ | .24 | .43^**^ | -.22 | -.05 |  | - | .44^***^ | .26^***^ | .37^***^ | -.03 | .11^*^ |
| 3. Exhibitionism |  | - | .43^**^ | .28^*^ | -.12 | .20 |  |  | - | .16^**^ | .37^***^ | -.10 | .14^**^ |
| 4. Exploitativeness |  |  | - | -.15 | .12 | .09 |  |  |  | - | -.02 | -.02 | .18^**^ |
| 5. Self Esteem |  |  |  | - | -.22 | .02 |  |  |  |  | - | .16^**^ | .08 |
| 6. RWA |  |  |  |  | - | .23 |  |  |  |  |  | - | .40^***^ |
| 7. SDO |  |  |  |  |  | - |  |  |  |  |  |  | - |

* *p* < .05. ** *p* < .01. *** *p* < .001.

Table S5.2

*Zero-order correlations between manifest variables separately for male and female respondents (Study 2)*

| Measure | Males (n = 193) | | | | | | |  | Females (n = 278) | | | | | | |
| --- | --- | --- | --- | --- | --- | --- | --- | --- | --- | --- | --- | --- | --- | --- | --- |
|  | 2 | 3 | 4 | 5 | 6 | 7 | 8. |  | 2 | 3 | 4 | 5 | 6 | 7 | 8. |
| 1. Narcissism | .87^***^ | .79^***^ | .61^***^ | .20^**^ | .22^**^ | .39^***^ | .35^***^ |  | .85^***^ | .77^***^ | .42^***^ | .31^***^ | -.06 | .31^***^ | .10 |
| 2.Authority | - | .54^***^ | .43^***^ | .29^***^ | .21^**^ | .27^***^ | .29^***^ |  | - | .50^***^ | .22^***^ | .35^***^ | -.00 | .24^***^ | .10 |
| 3.Exhibitionism |  | - | .40^***^ | .12 | .19^**^ | .31^***^ | .22^**^ |  |  | - | .25^***^ | .15^*^ | -.13^*^ | .23^***^ | .07 |
| 4.Exploitativeness |  |  | - | -.22^**^ | -.01 | .24^**^ | .18^*^ |  |  |  | - | -.13^*^ | -.07 | .27^***^ | .14^*^ |
| 5. Self Esteem |  |  |  | - | .16^*^ | .04 | .17^*^ |  |  |  |  | - | .19^**^ | .02 | .07 |
| 6. RWA |  |  |  |  | - | .40^***^ | .49^***^ |  |  |  |  |  | - | .25^***^ | .44^***^ |
| 7. SDO |  |  |  |  |  | - | .66^***^ |  |  |  |  |  |  | - | .50^***^ |
| 8. Prejudice |  |  |  |  |  |  | - |  |  |  |  |  |  |  | - |

* *p* < .05. ** *p* < .01. *** *p* < .001.

Table S5.3

*Zero-order correlations between manifest variables, separately for male (n = 118) and female (n = 171) respondents (Study 3)*

| Measure | Gender | 2. | 3. | 4. | 5. | 6. | 7. | 8. | 9. | 10. |
| --- | --- | --- | --- | --- | --- | --- | --- | --- | --- | --- |
| 1. Narcissism | M | .91^***^ | .81^***^ | .81^***^ | .52^***^ | .42^***^ | .05 | .12 | .34^***^ | .18 |
|  | F | .94^***^ | .82^***^ | .79^***^ | .38^***^ | .43^***^ | .27^***^ | -.03 | .38^***^ | .11 |
| 2. Authority | M | - | .57^***^ | .72^***^ | .36^***^ | .30^**^ | .13 | .13 | .27^**^ | .07 |
|  | F | - | .65^***^ | .71^***^ | .31^***^ | .38^***^ | .30^***^ | .04 | .36^***^ | .12 |
| 3. Exhibitionism | M |  | - | .62^***^ | .55^***^ | .36^***^ | -.10 | .10 | .27^**^ | .21^*^ |
|  | F |  | - | .57^***^ | .34^***^ | .30^***^ | .14 | -.17^*^ | .35^***^ | .10 |
| 4. Exploitativeness | M |  |  | - | .56^***^ | .48^***^ | -.14 | .10 | .41^***^ | .22^*^ |
|  | F |  |  | - | .48^***^ | .53^***^ | .01^***^ | -.03 | .37^***^ | .11 |
| 5. Psychopathy | M |  |  |  | - | .53^***^ | -.40^***^ | -.08 | .28^**^ | .30^**^ |
|  | F |  |  |  | - | .48^***^ | -.36^***^ | -.11 | .37^***^ | .33^***^ |
| 6. Machiavellianism | M |  |  |  |  | - | -.24^*^ | -.07 | .26^**^ | .32^***^ |
|  | F |  |  |  |  | - | -.21^**^ | .02 | .37^***^ | .29^***^ |
| 7. Self-Esteem | M |  |  |  |  |  | - | .22^*^ | -.04 | -.08 |
|  | F |  |  |  |  |  | - | .20^**^ | -.08 | -.10 |
| 8. RWA | M |  |  |  |  |  |  | - | .54^***^ | .39^***^ |
|  | F |  |  |  |  |  |  | - | .31^***^ | .35^***^ |
| 9. SDO | M |  |  |  |  |  |  |  | - | .70^***^ |
|  | F |  |  |  |  |  |  |  | - | .56^***^ |
| 10. Prejudice | M |  |  |  |  |  |  |  |  | - |
|  | F |  |  |  |  |  |  |  |  | - |

* *p* < .05, ** *p* < .01, *** *p* < .001.

Table 5.4

*Zero-order correlations between variables for male respondents (n = 149) in Study 4.*

| Measure | 2. | 3. | 4. | 5. | 6. | 7. | 8. | 9. | 10. | 11. |
| --- | --- | --- | --- | --- | --- | --- | --- | --- | --- | --- |
| 1. Narcissism | .12 | -.06 | .07 | .07 | .14^+^ | -.20* | -.17* | -.17* | .12 | -.06 |
| 2. Self Esteem | - | .23** | .11 | .28*** | .36*** | .16* | .25** | .45*** | .48*** | -.09 |
| 3. RWA |  | - | .49*** | .54*** | .06 | .20* | .30*** | .18* | -.06 | .30*** |
| 4. SDO |  |  | - | .24** | -.06 | .02 | .06 | .16^+^ | -.14^+^ | .40*** |
| 5. Collective Narcissism |  |  |  | - | .27*** | .14^+^ | .27*** | .25** | .13 | .21** |
| 6. Extraversion |  |  |  |  | - | .07 | .08 | .10 | .42*** | -.06 |
| 7. Agreeableness |  |  |  |  |  | - | .12 | .23** | .18* | .01 |
| 8. Conscientiousness |  |  |  |  |  |  | - | .34*** | .15^+^ | -.01 |
| 9. Emotional stability |  |  |  |  |  |  |  | - | .24** | .06 |
| 10. Openness |  |  |  |  |  |  |  |  | - | -.24** |
| 11. Prejudice |  |  |  |  |  |  |  |  |  | - |

^+^ *p* < .10, * *p* < .05, ** *p* < .01, *** *p* < .001.

Table S5.5

*Zero-order correlations between variables for female respondents (n = 612) in Study 4.*

| Measure | 2. | 3. | 4. | 5. | 6. | 7. | 8. | 9. | 10. | 11. |
| --- | --- | --- | --- | --- | --- | --- | --- | --- | --- | --- |
| 1. Narcissism | .10* | -.16** | .02 | -.08+ | .03 | -.29*** | -.16*** | -.15*** | -.01 | -.05 |
| 2. Self Esteem | - | -.10* | .03 | .01 | .34*** | .02 | .20*** | .38*** | .37*** | -.14*** |
| 3. RWA |  | - | .34*** | .50*** | -.09* | .09* | .17*** | .07+ | -.11** | .29*** |
| 4. SDO |  |  | - | .24*** | -.03 | -.02 | .09* | .02 | .02 | .32*** |
| 5. Collective Narcissism |  |  |  | - | .03 | .09* | .06 | .04 | .03 | .24*** |
| 6. Extraversion |  |  |  |  | - | .07 | .05 | .12** | .47*** | -.09* |
| 7. Agreeableness |  |  |  |  |  | - | .18*** | .30*** | .11** | -.07^+^ |
| 8. Conscientiousness |  |  |  |  |  |  | - | .28*** | .11** | .05 |
| 9. Emotional stability |  |  |  |  |  |  |  | - | .19*** | -.06 |
| 10. Openness |  |  |  |  |  |  |  |  | - | -.16*** |
| 11. Prejudice |  |  |  |  |  |  |  |  |  | - |

^+^ *p* < .10, * *p* < .05, ** *p* < .01, *** *p* < .001*.*

Table S5.6

*Results (standardized estimates) of models* ***only including*** ***male respondents*** *testing the associations of the narcissistic self-evaluation and non-narcissistic self-evaluation with SDO (free of RWA) and RWA (free of SDO) (Studies1-4) and ethnic prejudice (Studies 2-4)*

|  | Study 1  *n* = 49 | | Study 2  *n* = 193 | | Study 3  *n* = 118 | | Study 4  *n* = 149 | |
| --- | --- | --- | --- | --- | --- | --- | --- | --- |
| Associations | *β* | *p* | *β* | *p* | *β* | *p* | *β* | *p* |
| Narcissism - Self-Esteem | .414 | < .001 | .197 | .006 | .050 | .631 | .120 | .136 |
| Narc SE 🡪 SDO_f_ | .040 | .812 | .328 | < .001 | .288 | < .001 | .101 | .158 |
| Non-narc SE 🡪 SDO_f_ | .055 | .708 | -.080 | .259 | -.169 | .032 | -.021 | .779 |
| Narc SE 🡪 RWA_f_ | -.139 | .327 | .041 | .584 | -.093 | .216 | -.118 | .089 |
| Non-narc SE 🡪 RWA_f_ | -.164 | .244 | .137 | .055 | .244 | .002 | .191 | .006 |
| Narc SE 🡪 prejudice | / | / | .067 | .257 | -.068 | .322 | -.055 | .463 |
| Non-narc SE 🡪 Prejudice | / | / | .095 | .063 | -.056 | .408 | -.162 | .031 |
| SDO_f_ 🡪 Prejudice | / | / | .535 | < .001 | .714 | < .001 | .340 | < .001 |
| RWA_f_ 🡪 Prejudice | / | / | .246 | < .001 | .021 | .788 | .164 | .056 |

*Note*. Narc SE = Narcissistic Self-Evaluation; Non-narc SE = Non-narcissistic Self-Evaluation. The paths to SDO_f_ and RWA_f_ were calculated in two separate models which allowed us to estimate the associations for SDO, accounting for RWA (i.e. by including the path from RWA to SDO) and for RWA, accounting for SDO (i.e., by including the path from SDO to RWA).

Table S5.7

*Results (standardized estimates) of models only including* ***female respondents*** *testing the associations of the narcissistic self-evaluation and non-narcissistic self-evaluation with SDO (free of RWA) and RWA (free of SDO) (Studies1-4) and ethnic prejudice (Studies 2-4)*

|  | Study 1  *n* = 350 | | Study 2  *n* = 278 | | Study 3  *n* = 171 | | Study 4  *n* = 612 | |
| --- | --- | --- | --- | --- | --- | --- | --- | --- |
| Associations | *β* | *p* | *β* | *p* | *β* | *p* | *β* | *p* |
| Narc SE – Non-narc SE | .407 | < .001 | .306 | < .001 | .265 | < .001 | .095 | .019 |
| Narc SE 🡪 SDO_f_ | .201 | < .001 | .368 | < .001 | .466 | < .001 | .073 | .059 |
| Non-narc SE 🡪 SDO_f_ | -.064 | .212 | -.148 | .006 | -.281 | < .001 | .059 | .120 |
| Narc SE 🡪 RWA_f_ | -.189 | < .001 | -.229 | < .001 | -.281 | .002 | -.162 | < .001 |
| Non-narc SE 🡪 RWA_f_ | .201 | .001 | .251 | < .001 | .313 | < .001 | -.095 | .011 |
| Narc SE 🡪 prejudice | / | / | -.004 | .953 | -.054 | .436 | -.009 | .809 |
| Non-narc SE 🡪 Prejudice | / | / | -.003 | .953 | -.081 | .171 | -.129 | .001 |
| SDO_f_ 🡪 Prejudice | / | / | .415 | < .001 | .515 | < .001 | .258 | < .001 |
| RWA_f_ 🡪 Prejudice | / | / | .334 | < .001 | .201 | < .001 | .185 | < .001 |

*Note*. Narc SE = Narcissistic Self-Evaluation; Non-narc SE = Non-narcissistic Self-Evaluation. The paths to SDO_f_ and RWA_f_ were calculated in two separate models which allowed us to estimate the associations for SDO, accounting for RWA (i.e. by including the path from RWA to SDO) and for RWA, accounting for SDO (i.e., by including the path from SDO to RWA).

**Analyses using Rosenberg’s scale.**

We also conducted additional analyses for Study 4 in which we have used the Rosenberg self-esteem scale instead of using the single item measure of self-esteem as reported in the paper. The results for the Rosenberg scale are similar to the results for the single item measure.

Table S6

*Model results (standardized estimates) investigating the associations of narcissistic self-evaluation and non-narcissistic self-evaluation (Rosenberg self-esteem scale) with SDO (free of RWA) and RWA (free of SDO) and ethnic prejudice (Study 4).*

| Associations | *β* | *p* |
| --- | --- | --- |
| Narcissism – Self-Esteem | -.037 | .299 |
| Narc SE 🡪 SDO_f_ | .095 | .005 |
| Non-narc SE 🡪 SDO_f_ | .072 | .030 |
| Narc SE 🡪 RWA_f_ | -.153 | < .001 |
| Non-narc SE 🡪 RWA_f_ | .006 | .864 |
| Narc SE 🡪 Prejudice | -.037 | .276 |
| Non-narc SE 🡪 Prejudice | -.133 | < .001 |
| SDO_f_ 🡪 Prejudice | .280 | < .001 |
| RWA_f_ 🡪 Prejudice | .188 | < .001 |

*Note*. Narc SE = Narcissistic Self-Evaluation; Non-narc SE = Non-narcissistic Self-Evaluation. The paths to SDO_f_ and RWA_f_ were calculated in two separate models which allowed us to estimate the associations for SDO, accounting for RWA (i.e. by including the path from RWA to SDO) and for RWA, accounting for SDO (i.e., by including the path from SDO to RWA).
